# Detection of Bacterial *16S* rRNA and Identification of Four Clinically Important Bacteria by Real-Time PCR

**DOI:** 10.1371/journal.pone.0048558

**Published:** 2012-11-06

**Authors:** Robert J. Clifford, Michael Milillo, Jackson Prestwood, Reyes Quintero, Daniel V. Zurawski, Yoon I. Kwak, Paige E. Waterman, Emil P. Lesho, Patrick Mc Gann

**Affiliations:** 1 Multi-drug Resistant Organism Repository and Surveillance Network (MRSN), Walter Reed Army Institute of Research, Silver Spring, Maryland, United States of America; 2 Department of Wound Infections, Walter Reed Army Institute of Research, Silver Spring, Maryland, United States of America; University of Houston, United States of America

## Abstract

Within the paradigm of clinical infectious disease research, *Acinetobacter baumannii*, *Escherichia coli*, *Klebsiella pneumoniae*, and *Pseudomonas aeruginosa* represent the four most clinically relevant, and hence most extensively studied bacteria. Current culture-based methods for identifying these organisms are slow and cumbersome, and there is increasing need for more rapid and accurate molecular detection methods. Using bioinformatic tools, 962,279 bacterial *16S* rRNA gene sequences were aligned, and regions of homology were selected to generate a set of real-time PCR primers that target 93.6% of all bacterial 16S rRNA sequences published to date. A set of four species-specific real-time PCR primer pairs were also designed, capable of detecting less than 100 genome copies of *A. baumannii*, *E. coli*, *K. pneumoniae*, and *P. aeruginosa*. All primers were tested for specificity *in vitro* against 50 species of Gram-positive and –negative bacteria. Additionally, the species-specific primers were tested against a panel of 200 clinical isolates of each species, randomly selected from a large repository of clinical isolates from diverse areas and sources. A comparison of culture and real-time PCR demonstrated 100% concordance. The primers were incorporated into a rapid assay capable of positive identification from plate or broth cultures in less than 90 minutes. Furthermore, our data demonstrate that current targets, such as the *uidA* gene in *E.coli*, are not suitable as species-specific genes due to sequence variation. The assay described herein is rapid, cost-effective and accurate, and can be easily incorporated into any research laboratory capable of real-time PCR.

## Introduction

In clinical microbiology laboratories, traditional culture based techniques remain the primary methodology used for identifying bacterial isolates. These methods are time-consuming, can involve multiple biochemical tests, and can be expensive, particularly for fastidious organisms. In recent years, the introduction of automated identification instruments has resulted in greater reliability, but discrepancies between the various platforms have been reported [1]–[3]. Furthermore, many research laboratories lack the funds necessary to invest in these instruments, and often rely on labor-intensive biochemical characterization or *16S* rRNA sequencing to confirm species type.

Despite significant advances in molecular biology, molecular methods for species identification have not achieved widespread use, with the possible exception of the Biodefense community [4]. Factors such as allelic variation within target genes, cross-reaction with other species [5] and the lack of experienced molecular technicians have all contributed to their relative scarcity. However, despite these limitations, molecular methods can provide significant advantages over phenotypic-based methods, including rapid turnaround time, scalability, and high sensitivity [5]. Furthermore, using appropriate bioinformatic tools and careful primer design, many of the limitations outlined above can be mitigated considerably.

Even prior to recent global calls to help combat the spread of carbapenem-resistant *Enterobacteriaceae* (CRE), particularly *E.coli* and *K. pneumoniae* [6]–[8], research on CRE, multi-drug resistant *A. baumannii* and *P. aeruginosa*, and methicillin-resistant *Staphylococcus aureus* (MRSA) has been prolific, as befits their status as the most important nosocomial pathogens. A number of conventional PCR and real-time PCR assays have been developed targeting these organisms [9]–[13], but a comprehensive panel that can target all five clinically important species is lacking.

We recently published a highly sensitive and specific real-time PCR assay for the detection of MRSA [14]. This report adds an additional four real-time PCR primers, designed using a combination of *in silico* and *in vitro* methodologies that are highly specific to the four most common nosocomial pathogens. This primer set will allow researchers to rapidly identify these pathogens directly from culture in less than 90 minutes with a high degree of accuracy and sensitivity. We also describe a novel bacterial *16S* rRNA real-time PCR primer designed from the alignment of 962,279 bacterial *16S r*RNA gene sequences, which will amplify a product from the *16S* rRNA gene in 93.6% of all bacterial *16S* rRNA genes published to date. The combination of *16S* rRNA gene detection with species-specific primers can greatly reduce the effort required in screening large populations of unknown organisms, with samples negative for *16S* rRNA being excluded from further testing.

## Materials and Methods

### Primer Design

Primer characteristics are presented in Table 1. Unless noted otherwise, all primers were designed using Primer Express version 2.0 (Applied Biosystems, Carlsbad, CA). All primers were designed to have an optimal annealing temperature of 56°C, and real-time PCR reactions were performed in 20 µl volumes with 200 nM primers. Where appropriate, alignment of individual gene sequences was performed using MegAlign version 10.0.1 (DNAStar Inc, Madison, WI).

**Table 1:** Primer characteristics.

Name^1^	Gene	Target species	Sequence (5′ to 3′)	Eff (%)^2^	Length^3^
U16SRT-F	*16S*	variable	ACTCCTACGGGAGGCAGCAGT	>96.4%	180
U16SRT-R			TATTACCGCGGCTGCTGGC		
secERT-F		*A.baumannii*	GTTGTGGCTTTAGGTTTATTATACG		
secERT-R	*secE*	*A. nosocomialis*	AAGTTACTCGACGCAATTCG	99.4	94
secERT-Probe		*A. pitii*	ACCCATCAAGGTAAAGGCTTCGTTCG		
yccTRT-F		*Escherichia coli*	GCATCGTGACCACCTTGA		
yccTRT-R	*yccT*	*Shigella* spp.	CAGCGTGGTGGCAAAA	98.1	59
yccTRT-Probe			TGCATTATGTTTGCCGGTATCCG		
gltART-F	*gltA*	*K. pneumoniae*	ACGGCCGAATATGACGAATTC	97.1	68
gltART-R			AGAGTGATCTGCTCATGAA		
ecfXRT-F	*ecfX*	*P.aeruginosa*	AGCGTTCGTCCTGCACAAGT	93.8	81
ecfXRT-R			TCCACCATGCTCAGGGAGAT		

1 Probe sequences were generated for the *secE* and *yccT* real-time PCR primers, but no *in vitro* testing was performed. The probe sequences are provided here for the benefit of researchers wishing to perform multiplex real-time PCR reactions using these primers. All primers have an optimal annealing temperature of 56°C.

2 Primer efficiency was calculated from the slope and intercept of the trendline produced following amplification of serial dilutions of genomic DNA from the ATCC strains of each species, as described previously [14].

3 Amplicon size in base pairs.

### 16S Real-time PCR Primers

*16S* rRNA real-time PCR primers were designed manually from a consensus sequence based on an alignment of 962,279 bacterial *16S* rRNA gene sequences obtained from the Ribosomal Database Project release 10 [15]. To derive the consensus sequence, nucleotide frequencies were determined at each position in the alignment using a custom script in Perl ver 5.8.8 running on the Red Hat Linux operating system; positions where the majority of sequences had a gap were excluded. Regions of the consensus sequence where the majority nucleotide was present in >90% of the sequences were used for primer design. A synopsis of each nucleotide frequency is presented as supplemental material (Table S1).

### *E. coli* and *A. baumannii* – Specific Primers

To identify genes specific to *E. coli* and *A. baumannii*, annotated complete genome sequences for 38 *E. coli* strains, and 8 completed and 52 draft *A. baumannii* genomes were selected (Table S2 and S3). In addition, the two draft genomes of *A. nosocomialis* (RUH 2624 and NCTC 8102) and the four draft genomes of *A. pitii* (SH024, D499, DSM 21653, and DSM 9306) were used to ensure that all *Acinetobacter*-specific primers would also amplify products from these two species. For each protein in a strain, BLASTP [16] was used to find the best match to a protein family in the HOGENOM release 5 database; orthologous proteins from different strains show the best match to the same HOGENOM protein family [17]. Genes that are not single copy (that is, two or more proteins from the same strain are members of the same HOGENOM protein family) were excluded from further analysis. In addition genes for which the nucleotide length of any ortholog varies from the mean gene length by more than 5% were excluded in order to restrict the analysis to those genes that were highly conserved. For the genes that met these criteria and had the fewest variable positions, BLASTN analysis was performed against the NCBI non-redundant nucleotide database using a gene sequence randomly selected from the final species-specific gene targets.

Four genes showed no significant match to non-*Escherichia* species: HBG518163 (acid chaperone protein, *hde*A), HBG636731 (predicted secreted protein, function unknown, *ynfB*), HBG473849 (conserved protein, function unknown, *yccT*), and HBG758393 (function unknown, DUF1418 family, *ybjC*). Nineteen genes showed no significant match to non-*Acinetobacter* species (Table S4), and the top three candidates, based on total gene length and number of mismatches between all genomes; HBG594899 (*rpiN,* 50S ribosomal protein L14), HBG701403 (*secE,* preprotein translocase, SecE subunit), and HBG752450 (*scpB,* segregation and condensation protein B) were selected for further analysis.

Starting with a multiple sequence alignment, a custom Perl script was used to identify positions within each gene that are invariant among all strains. With *E. coli*, two of the four genes (*hdeA* and *yccA*) contained regions >100 bp that were suitable for primer design. All three genes from the *Acinetobacter* analysis contained extensively conserved regions suitable for primer design.

### *K. pneumoniae* and *P. aeruginosa* – Specific Primers

The limited number of published genome sequences available for *K. pneumoniae* and *P. aeruginosa* precluded extensive bioinformatic analysis. Therefore, the top two candidate genes from both species (*K. pneumoniae*; *gltA* and *khe*; *P. aeruginosa; ecfX* and *23S*) were selected based on conserved gene sequences from the available genomes.

### Validation of Real-time PCR Primers

All primer sets were tested for sensitivity, optimal annealing temperature and primer efficiency as previously described [18], [19]. Briefly, high quality gDNA was was extracted using the GeneJET Genomic DNA Purification Kit (Fermentas Inc, Glen Burnie, MD, USA) with an additional bead-beating step to maximize lysis. DNA was quantified using both a Nanodrop 2000 (Nanodrop, Wilmington, DE, USA) and Qubit 2.0 Fluorometer (Life technologies, Grand Island, NY, USA).

Genome copies of DNA were calculated using the following formula:

Size of genome (in bp) × 650 Daltons/bp = molecular weight of Genome in g/mol.

# copies of genome in 1 ng of DNA = (1×10^−9^ g ÷ Mw of genome)×6.02×10^23^ molecules/mole (Avogadro’s number).

To get 10^8^ copies in 2 µl = (10^8^ ÷ # copies of genome in 1 ng)/2.

Serial dilutions of DNA from 10^8^ to 10^2^ copies were prepared and tested in duplicate with each primer set to calculate primer efficiency and sensitivity.

Primers were tested using two different instruments, the Roche Light Cycler 480 II (LC 480 II) with SYBR Green I Master Mix (Roche Applied Sciences, Indianapolis, IN), and the BioRad CFX96 with SsoAdvanced SYBR Green supermix (Bio-Rad Laboratories, Hercules, CA). Each primer was tested for specificity by two methods. First, the primers were tested against genomic DNA extracted from a panel of American Type Culture Collection strains (ATCC, Manassas, VA, USA) and clinical isolates representing fifty different bacterial species, including closely related members from the same genus (Table S5). Secondly, primers were tested against 200 clinical isolates of each species, identified to the species level using three automated identification systems; the Vitek 2 (bioMerieux, Durham, NC), the BD Pheonix (Diagnostics Systems, Sparks, MD), and the Microscan Walkway (Siemens Healthcare Diagnostics Inc, Deerfield, IL), selected from a large repository of isolates (>10,000 strains) collected between 2002 to 2012 from 23 different facilities in the United States of America, Europe, Asia and the Middle East. Pulsed-field gel electrophoresis (PFGE) indicated that the selected clinical isolates represented a variety of different pulse-types (unpublished results).

### High throughput Testing of Clinical Isolates

The primers were incorporated into a 96-well plate assay to allow high throughput testing of multiple clinical isolates. All bacterial isolates were cultured overnight on Blood Agar plates at 37°C, and all samples were prepared in a BioSafety hood in a certified BSL2 laboratory. Single colonies from an overnight culture of each isolate were re-suspended in 200 µl of sterile, ultra-pure water and mixed by vortexing. 10 µl of the resulting suspension (or 10 µl taken directly from an overnight broth culture) was added to 20 µl of Lyse-and-Go reagent (Thermo Scientific, Waltham, MA) in 96-well plates, and run in a thermal cycler using the manufacturer’s protocol for the isolation of total genomic DNA. Isolates were held at 80°C for 15 minutes at the end of the program to maximize lysis, and 2 µl of the resulting lysate was used directly for real-time PCR. Leftover bacterial DNA in Lyse-and-Go reagent was stored at −20°C, and no reduction in real-time PCR amplification was evidenced after 9 months (data not shown). Appropriate positive (ATCC Type strains for each species), negative (two ATCC type strains from species other than the target organism), and no template controls (ultra-pure water) were incorporated onto every plate. Cycling parameters were 95°C for 5 minutes, followed by 40 cycles of 95°C for 10 seconds and 56°C for 10 seconds. A melting curve analysis was included at the end of every program to assist in data analysis. Quantification cycle (Cq; CFX96) and crossing threshold (Ct; LC 480 II) values were calculated automatically using instrument software.

**Figure 1:**
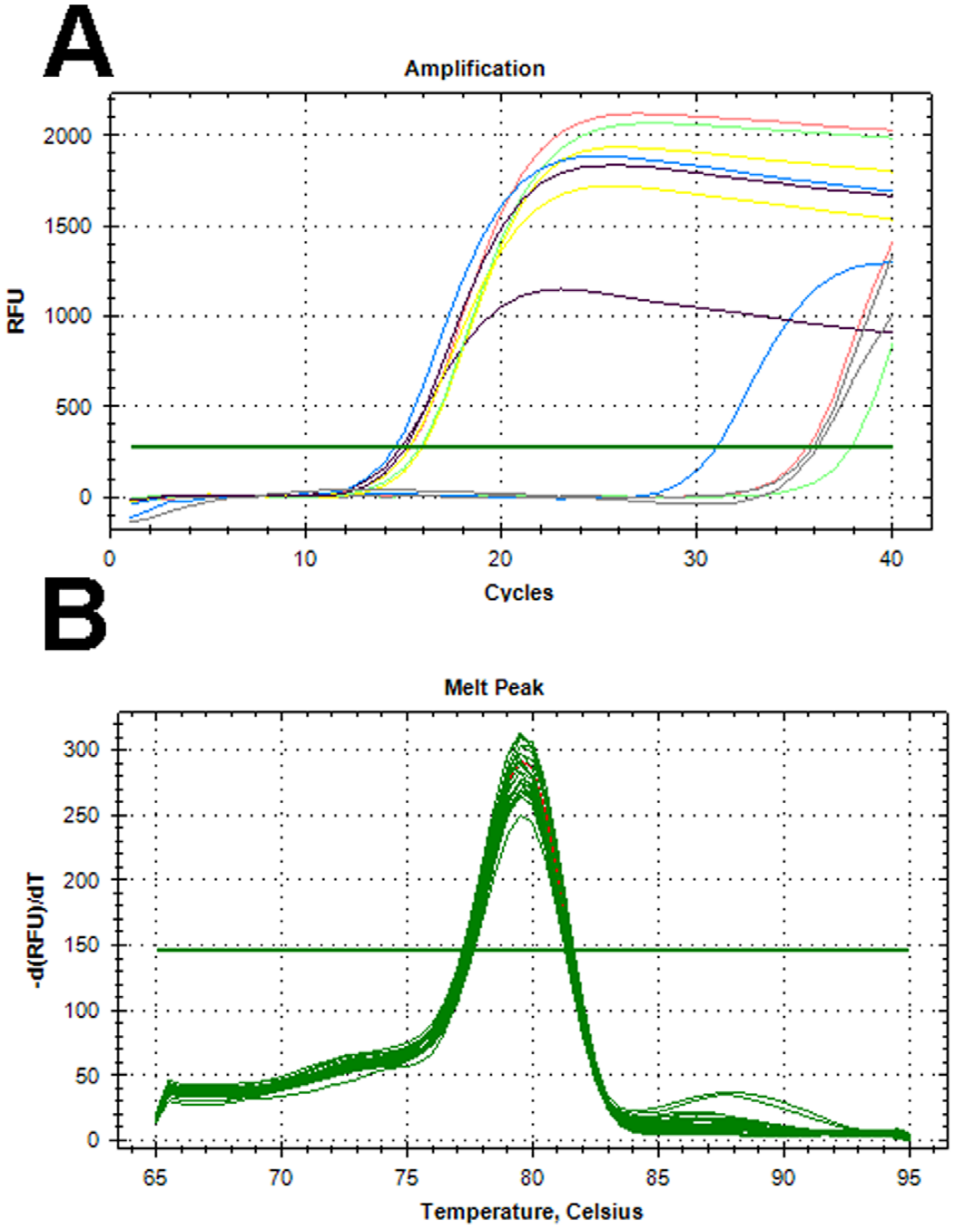
Amplification and melting curves for selected isolates. (**A**) Amplification curves of three of the eight *E. coli* isolates that were *uidA*-negative, but *yccT*-positive; MRSN 1628 (Red line), MRSN 1681 (Blue Line), and MRSN 7544 (Green Line). MRSN 7851 (Yellow Line) was both *uidA* and *yccT*-positive. *E.coli* ATCC 35218 (Black line) was used as a positive control and *K. pneumoniae* ATCC 1706 (Grey Line) was used a negative control. Each amplification curve is one representative sample from quintuplicate experiments. (**B**) Melting curve analysis of the amplicons produced by the *yccT* primer pair from 30 clinical isolates of *E. coli* demonstrating a highly conserved sequence in all strains. Melting curve of the *yccT* amplicon from *E. coli* ATCC 35218 is shown in red. All 30 isolates represented diverse pulse-types as determined by PFGE. RFU - Relative Fluorescent Units; -d(RFU)dt – Relative change in RFU over time (in seconds).

## Results and Discussion

### 16S rRNA Real-time PCR Primers

Analysis of the alignment of 962,279 bacterial *16S* rRNA gene sequences revealed considerable nucleotide variation between species (Table S1). However, two regions were identified that were suitable for primer design, where the nucleotide sequence was highly conserved in >90% of all rRNA sequences. Forward and reverse primers were manually designed, with the nucleotide sequence of the forward primer being conserved in 98.5% of *16S* rRNA sequences, and the nucleotide sequence in the reverse primer being conserved in 93.8% (Table S1).

Primers were tested against serial dilutions of genomic DNA from *A. bauamnnii* ATCC 19606, *E. coli* ATCC 35218, and *P. aeruginosa* ATCC 27853. The primers were reproducibly capable of detecting <100 copies of genomic DNA, and primer efficiency was 96.4%, 98.2% and 97.9% respectively, with an R^2^>99%. The primers successfully amplified a product from the 50 ATCC and clinical strains (Table S5), providing *in vitro* support for the *in silico* primer design method employed. No amplification was seen from no-template negative controls, or from DNA extracted from 15 clinical isolates of *Candida parapsilosis* and *C. albicans* (data not shown).

The *16S* rRNA gene is a frequent target for many assays, and universal PCR primers are routinely used for species identification [20]. A number of real-time primers for this gene have also been developed, but they either contain degenerate nucleotides [21], or produce long amplicons that are not suitable for optimal real-time PCR [22]. The primer set described here overcomes both these limitations, and has proved a valuable tool in detecting bacterial contamination of environmental swabs (manuscript in preparation), as a positive control for bacterial lysis, confirming that plasmid DNA is free of contaminating genomic DNA, and determining if a colony in a mixed culture is bacterial.

### Species-specific Real-time PCR Primers

Five sets of primers were tested for sensitivity and specificity for *A. baumannii;* three primers were generated using the genome alignment algorithm (*rpiN, secE* and *scpB*), one primer was designed in-house from an alignment of 54 *ompA* sequences, and the fifth primer, also targeting the *ompA* gene, has been published previously [11]. *OmpA* was included as a potential target as this gene has been used as a target for *A. baumannii*-specific primers in previous publications [11]. However, though *ompA* was identified as a potential target using our algorithm, sequence variation among *A. baumannii*, *A. nosocomialis* and *A. pitii*, as well as variation in the protein length, resulted in this gene being flagged as unsuitable as an *Acinetobacter*-specific target. All five primers did not cross-react with other bacterial species (Table S5), including *A. lwoffi*. Primer efficiency at 56°C, based on serial dilutions of *A. baumannii* AB0057 and *A. baumannii* ACICU, ranged from 97.2% (*rpiN*) to 99.4% (*secE*), with R^2^>99%, and all primers were reproducibly capable of detecting <100 genome copies of DNA. Furthermore, all five primers successfully amplified products from the 200 clinical test isolates. The primer pair targeting the *secE* gene consistently provided greater sensitivity than all other primers at an annealing temperature of 56°C, and was therefore selected as the optimal candidate for identifying *A. baumannii*. *In silico* analysis of the *secE* primer with the draft *A. nosocomialis* and *A. pitii* genomes demonstrated that these primers would also amplify a product from these two species.

Four sets of primers were tested for sensitivity and specificity for *E.coli*; two primers were generated using the genome alignment algorithm (*hdeA* and *yccT*), one primer was designed from an alignment of 38 *uidA* genes (Table S2), and a fourth primer, also targeting the *uidA* gene, has been described previously [7], [18]. *UidA* was included as this gene has been used as a species-specific target in a number of previous publications [10], [12], [23]–[25], but it did not meet the criteria demanded by the species-specific algorithm in this study. This was confirmed by both *in silico* and *in vitro* testing. An alignment of 38 *E.coli* genomes indicated that published *uidA* primer sequences [10], [12] have 3 nucleotide variations in the forward primer and 1 nucleotide variation in the reverse primer when aligned with some *E.coli uidA* gene sequences. When tested against the 200 clinical isolates, the primers failed to amplify any product from 8 strains (Figure 1a). A second set of *uidA* primers was designed, targeting a conserved region of the 38 *uidA* gene sequences. However, no amplification was again observed from 7 of the same 8 strains that were *uidA* negative from the first primer pair. One of these 7 isolates amplified a product after 28 cycles, indicative of inefficient primer annealing, most likely due to primer/target mismatches within the *uidA* gene.

The final two primers, targeting the *yccT* and *hdeA* genes, were identified as the most promising candidates based on the algorithm used in this study. Primer efficiency was 98.1% and 97.4% respectively, with R^2^>99%. However, 9 of the 200 clinical isolates failed to amplify a product with the *hdeA* primer, including 3 of the 8 *uidA*-negative isolates. In contrast, the *yccT* primer successfully amplified a highly conserved product from all 200 isolates (Figure 1b), and was therefore selected as the final *E. coli-*specific primer. As expected, due to the very high homology between *E. coli* and *Shigella* strains [26], the *yccT* primer set also amplified a product from *Shigella flexneri* ATCC 12022, but did not cross-react with any other species (Table S5).

Despite 38 sequenced genomes of *E. coli*, developing an *E. coli*-specific primer pair is challenging. We have shown that current targets, such as the *uidA* gene are not suitable due to considerably allelic variation, particularly among *E. coli* clinical isolates from diverse regions. The remarkable diversity among *E. coli* strains is also highlighted in the bioinformatic approach that we used, where just 2 candidate genes passed all of the criteria employed, and just a single gene, *yccT*, was eventually successful *in vitro*.

The paucity of completed genomic sequences for both *K. pneumoniae* and *P. aeruginosa* limited the application of our algorithm to just a single genome for *P. aeruginosa* with an additional 4 whole genome shotgun sequences, and 5 genomes for *K. pneumoniae*.

BLAST analysis suggested a potential cross-reaction between the *K. pneumoniae khe* primer set and *Citrobacter fruendii*, and this was confirmed *in vitro*. However, the *gltA* primer set demonstrated no cross-reactivity with other species (Table S5), a primer efficiency of 97.1%, and successfully amplified a product from all 200 clinical isolates of *K. pneumoniae*.

Both sets of *P. aeruginosa* primers demonstrated no cross-reactivity with other species, including *P. flourescens*, *P. stutzeri* and *P. putida*. However, when tested against a panel of 200 clinical isolates of *P. aeruginosa* only the *ecfX* primer successfully amplified a product from all strains, with 12 strains failing to generate a product with the *23S* rRNA primer set. The *ecfX* primer set demonstrated a primer efficiency of 93.8% with an R^2^ value of 99.1%.

Species-specific primers targeting the *ecfX* gene in *P. aeruginosa* have been published for both conventional [27], [28] and real-time PCR assays [9], [29]. Our results support the continued use of this gene as a target for *P. aeruginosa*-specific primers, at least until further full genome sequences of this strain become available. In contrast, species-specific primers for *K. pneumoniae* are less well described, and primarily involve detecting targets that are specific to certain strains, such as the ST258 clone [30], or those with the hypermucoviscosity phenotype [31]. In addition, a number of conventional PCR assays to detect *K. pneumoniae* have been described [13], [32], though to our knowledge, no assay described to date uses *gltA* as the target gene.

### Conclusion

We describe a set of real-time PCR primers, designed to have the same optimal annealing temperature, and displaying high specificity for four clinically important pathogens. The primers are well suited for high-throughput testing of isolates, with results available in less than 90 minutes from bacterial colonies or overnight broth cultures. We also demonstrate the power of bioinformatics in designing optimal primer sequences, and provide a novel *16S* rRNA real-time PCR primer designed from the alignment of over 960,000 bacterial *16S* rRNA sequences. The primers described herein have been an invaluable addition to our surveillance network, and have demonstrated a 100% concordance with traditional phenotypic identification systems.

## Supporting Information

Table S1 **Nucleotide frequencies at each position from an alignment of 962,279** ***16S*** **rRNA sequences.**
(DOCX)

Table S2 **List of assembled** ***E. coli*** **genomes used in the design of** ***E.coli*** **-specific primers.**
(DOCX)

Table S3 **Complete and draft** ***A. baumannii*** **genomes used the design of** ***Acinetobacter*** **-specific primers.**
(DOCX)

Table S4 **The top 28 gene targets for designing** ***Acinetobacter*** **-specific primers.**
(DOCX)

Table S5 **Bacterial species used for specificity testing of species-specific primers.**
(DOCX)
