# Supplementary material for: Detection of Bacterial 16S rRNA and Identification of Four Clinically Important Bacteria by Real-Time PCR
Source: PLoS One. 2012 Nov 6;7(11):e48558. doi: 10.1371/journal.pone.0048558 (PMC3490953; doi:10.1371/journal.pone.0048558)
Supplement: Table S1 — Nucleotide frequencies at each position from an alignment of 962,279 16S rRNA sequences. (DOCX) [file pone.0048558.s001.docx]

**Supplemental Table S1. Nucleotide frequencies at each position from an alignment of 962,279 *16S* rRNA sequences**

|  | | | **Nucleotide^2^** | | |  | | |
| --- | --- | --- | --- | --- | --- | --- | --- | --- |
| **Position^1^** | **None** | **A** | | **C** | **G** | | **T** | **Majority^3^** |
| 642 | 0.1497 | 0.2194 | | 0.0015 | 0.6279 | | 0.0014 | G |
| 648 | 0.1286 | 0.691 | | 0.001 | 0.0233 | | 0.1559 | A |
| 654 | 0.1261 | 0.0007 | | 0.3556 | 0.001 | | 0.5164 | T |
| 683 | 0.1235 | 0.0078 | | 0.0051 | 0.8558 | | 0.0076 | G |
| 690 | 0.1206 | 0.8695 | | 0.0009 | 0.0026 | | 0.0063 | A |
| 698 | 0.1174 | 0.8759 | | 0.0019 | 0.0031 | | 0.0015 | A |
| 703 | 0.1148 | 0.0004 | | 0.8802 | 0.0009 | | 0.0035 | C |
| 710 | 0.112 | 0.0011 | | 0.0022 | 0.8773 | | 0.0072 | G |
| 715 | 0.109 | 0.0004 | | 0.8738 | 0.0007 | | 0.0159 | C |
| 722 | 0.1064 | 0.0008 | | 0.004 | 0.0012 | | 0.8875 | T |
| 734 | 0.104 | 0.0803 | | 0.001 | 0.8019 | | 0.0125 | G |
| 741 | 0.1009 | 0.0059 | | 0.001 | 0.8829 | | 0.0091 | G |
| 747 | 0.0986 | 0.0007 | | 0.8899 | 0.0014 | | 0.0092 | C |
| 754 | 0.0965 | 0.0063 | | 0.0009 | 0.8597 | | 0.0365 | G |
| 762 | 0.0941 | 0.0437 | | 0.0005 | 0.8595 | | 0.0021 | G |
| 771 | 0.0915 | 0.0094 | | 0.8559 | 0.0161 | | 0.0268 | C |
| 777 | 0.0901 | 0.3194 | | 0.0015 | 0.5879 | | 0.001 | G |
| 782 | 0.0869 | 0.0018 | | 0.0346 | 0.2013 | | 0.6751 | T |
| 788 | 0.0811 | 0.003 | | 0.0005 | 0.9149 | | 0.0003 | G |
| 793 | 0.0793 | 0.0003 | | 0.9034 | 0.0113 | | 0.0055 | C |
| 811 | 0.0781 | 0.0164 | | 0.4987 | 0.0016 | | 0.405 | C |
| 823 | 0.0772 | 0.0007 | | 0.0022 | 0.0002 | | 0.9194 | T |
| 838 | 0.0757 | 0.8386 | | 0.0036 | 0.0021 | | 0.0797 | A |
| 851 | 0.0748 | 0.921 | | 0.0019 | 0.0015 | | 0.0006 | A |
| 859 | 0.0704 | 0.0047 | | 0.6153 | 0.0172 | | 0.2923 | C |
| 869 | 0.0697 | 0.9143 | | 0.001 | 0.0142 | | 0.0006 | A |
| 876 | 0.0675 | 0.0005 | | 0.9306 | 0.0002 | | 0.0011 | C |
| 887 | 0.0651 | 0.9314 | | 0.0007 | 0.0018 | | 0.0008 | A |
| 892 | 0.0575 | 0.0003 | | 0.0023 | 0.0002 | | 0.9397 | T |
| 896 | 0.0556 | 0.0008 | | 0.0003 | 0.9424 | | 0.0008 | G |
| 900 | 0.0545 | 0.0002 | | 0.9438 | 0.0002 | | 0.0012 | C |
| 907 | 0.0533 | 0.9396 | | 0.0016 | 0.0024 | | 0.0027 | A |
| 924 | 0.0597 | 0.9346 | | 0.0005 | 0.0018 | | 0.0032 | A |
| 929 | 0.0484 | 0.0007 | | 0.0003 | 0.948 | | 0.0024 | G |
| 933 | 0.0463 | 0.0004 | | 0.0136 | 0.0004 | | 0.9391 | T |
| 940 | 0.0442 | 0.0564 | | 0.8914 | 0.0015 | | 0.0063 | C |
| 947 | 0.0443 | 0.0035 | | 0.0013 | 0.9484 | | 0.0025 | G |
| 952 | 0.0378 | 0.9173 | | 0.0091 | 0.0186 | | 0.017 | A |
| 958 | 0.0329 | 0.561 | | 0.001 | 0.4019 | | 0.0029 | A |
| 961 | 0.0265 | 0.0013 | | 0.8997 | 0.0668 | | 0.0056 | C |
| 962 | 0.0169 | 0.002 | | 0.0023 | 0.9696 | | 0.009 | G |
| 963 | 0.0198 | 0.3565 | | 0.1099 | 0.4995 | | 0.0141 | G |
| 964 | 0.0202 | 0.4486 | | 0.1154 | 0.1524 | | 0.2631 | A |
| 965 | 0.0207 | 0.4626 | | 0.2246 | 0.2427 | | 0.0491 | A |
| 966 | 0.0214 | 0.5361 | | 0.0964 | 0.2408 | | 0.1051 | A |
| 967 | 0.022 | 0.2321 | | 0.2611 | 0.3691 | | 0.1155 | G |
| 968 | 0.0225 | 0.3756 | | 0.0921 | 0.4026 | | 0.1069 | G |
| 969 | 0.0233 | 0.1212 | | 0.413 | 0.2034 | | 0.2387 | C |
| 970 | 0.0237 | 0.1815 | | 0.2545 | 0.3573 | | 0.1826 | G |
| 971 | 0.0245 | 0.2992 | | 0.2138 | 0.2531 | | 0.2089 | A |
| 972 | 0.0251 | 0.1953 | | 0.1539 | 0.3921 | | 0.2331 | G |
| 973 | 0.0262 | 0.2379 | | 0.089 | 0.4517 | | 0.1949 | G |
| 974 | 0.0269 | 0.3109 | | 0.2254 | 0.2316 | | 0.2049 | A |
| 975 | 0.0402 | 0.1387 | | 0.2339 | 0.2877 | | 0.2991 | T |
| 976 | 0.0416 | 0.1011 | | 0.2839 | 0.2412 | | 0.332 | T |
| 977 | 0.0778 | 0.0863 | | 0.1055 | 0.301 | | 0.4292 | T |
| 978 | 0.082 | 0.1184 | | 0.173 | 0.2289 | | 0.3975 | T |
| 979 | 0.1 | 0.1029 | | 0.177 | 0.3487 | | 0.2712 | G |
| 980 | 0.1018 | 0.1319 | | 0.2936 | 0.1975 | | 0.2751 | C |
| 981 | 0.1091 | 0.0715 | | 0.2323 | 0.2155 | | 0.3714 | T |
| 982 | 0.1108 | 0.0978 | | 0.2732 | 0.2285 | | 0.2896 | T |
| 983 | 0.1145 | 0.0853 | | 0.2867 | 0.1914 | | 0.3217 | T |
| 984 | 0.1174 | 0.1307 | | 0.1847 | 0.2355 | | 0.3314 | T |
| 985 | 0.1369 | 0.1171 | | 0.2664 | 0.1662 | | 0.313 | T |
| 986 | 0.1436 | 0.1237 | | 0.0666 | 0.3412 | | 0.3245 | G |
| 987 | 0.2092 | 0.0914 | | 0.2067 | 0.3331 | | 0.1595 | G |
| 988 | 0.2245 | 0.2778 | | 0.0714 | 0.2189 | | 0.2072 | A |
| 990 | 0.3586 | 0.1049 | | 0.0456 | 0.3783 | | 0.1126 | G |
| 2281 | 0.0144 | 0.9767 | | 0.0035 | 0.0048 | | 0.0005 | A |
| 2282 | 0.0139 | 0.0031 | | 0.0675 | 0.9141 | | 0.0012 | G |
| 2287 | 0.0139 | 0.0011 | | 0.4107 | 0.0004 | | 0.5738 | T |
| 2297 | 0.0131 | 0.0042 | | 0.0014 | 0.9247 | | 0.0565 | G |
| 2301 | 0.0127 | 0.0009 | | 0.0002 | 0.9855 | | 0.0005 | G |
| 2305 | 0.0123 | 0.0002 | | 0.9857 | 0.0004 | | 0.0013 | C |
| 2312 | 0.0118 | 0.0386 | | 0.0004 | 0.9487 | | 0.0003 | G |
| 2318 | 0.0115 | 0.417 | | 0.0972 | 0.4579 | | 0.0163 | G |
| 2326 | 0.0111 | 0.986 | | 0.0012 | 0.0015 | | 0.0002 | A |
| 2332 | 0.0111 | 0.0094 | | 0.9709 | 0.0003 | | 0.0083 | C |
| 2338 | 0.0105 | 0.0008 | | 0.0002 | 0.9801 | | 0.0083 | G |
| 2347 | 0.0103 | 0.0009 | | 0.0001 | 0.9884 | | 0.0002 | G |
| 2350 | 0.0099 | 0.0006 | | 0.0052 | 0.9835 | | 0.0007 | G |
| 2354 | 0.0098 | 0.0029 | | 0.0034 | 0.0024 | | 0.9815 | T |
| 2361 | 0.0095 | 0.0005 | | 0.0011 | 0.9827 | | 0.0062 | G |
| 2370 | 0.0091 | 0.9307 | | 0.0582 | 0.0015 | | 0.0005 | A |
| 2382 | 0.009 | 0.0009 | | 0.0003 | 0.9891 | | 0.0007 | G |
| 2389 | 0.0087 | 0.0005 | | 0.002 | 0.0248 | | 0.9639 | T |
| 2396 | 0.0085 | 0.9894 | | 0.0006 | 0.001 | | 0.0004 | A |
| 2410 | 0.0087 | 0.9805 | | 0.0012 | 0.0026 | | 0.0069 | A |
| 2422 | 0.0082 | 0.008 | | 0.7951 | 0.0029 | | 0.1858 | C |
| 2427 | 0.008 | 0.5878 | | 0.0004 | 0.4031 | | 0.0005 | A |
| 2434 | 0.0078 | 0.0007 | | 0.9179 | 0.0017 | | 0.0718 | C |
| 2438 | 0.0077 | 0.0961 | | 0.0538 | 0.8004 | | 0.0419 | G |
| 2442 | 0.0075 | 0.0009 | | 0.0024 | 0.0003 | | 0.9888 | T |
| 2445 | 0.0074 | 0.2777 | | 0.0738 | 0.6374 | | 0.0036 | G |
| 2461 | 0.0073 | 0.2016 | | 0.0049 | 0.708 | | 0.0781 | G |
| 2465 | 0.0071 | 0.1407 | | 0.0402 | 0.804 | | 0.0079 | G |
| 2469 | 0.007 | 0.2517 | | 0.1627 | 0.0627 | | 0.5158 | T |
| 2479 | 0.2676 | 0.6375 | | 0.0011 | 0.0906 | | 0.0031 | A |
| 2497 | 0.0069 | 0.991 | | 0.0002 | 0.0016 | | 0.0003 | A |
| 2501 | 0.0067 | 0.0256 | | 0.6969 | 0.002 | | 0.2688 | C |
| 2505 | 0.0065 | 0.0192 | | 0.8536 | 0.0902 | | 0.0303 | C |
| 2511 | 0.0064 | 0.0019 | | 0.0039 | 0.0005 | | 0.9873 | T |
| 2520 | 0.0063 | 0.289 | | 0.0011 | 0.6912 | | 0.0121 | G |
| 2544 | 0.0064 | 0.0008 | | 0.9583 | 0.0139 | | 0.0205 | C |
| 2548 | 0.0061 | 0.0003 | | 0.9895 | 0.0001 | | 0.0038 | C |
| 2553 | 0.006 | 0.0077 | | 0.4428 | 0.0316 | | 0.5119 | T |
| 2557 | 0.0059 | 0.2946 | | 0.1327 | 0.145 | | 0.4217 | T |
| 2564 | 0.0059 | 0.1804 | | 0.0704 | 0.2916 | | 0.4515 | T |
| 2570 | 0.0058 | 0.287 | | 0.1693 | 0.1544 | | 0.3834 | T |
| 2574 | 0.0056 | 0.7828 | | 0.0385 | 0.118 | | 0.055 | A |
| 2579 | 0.0054 | 0.0173 | | 0.3544 | 0.6187 | | 0.0041 | G |
| 2606 | 0.0051 | 0.3916 | | 0.1047 | 0.003 | | 0.4955 | T |
| 2612 | 0.0049 | 0.0457 | | 0.2972 | 0.4676 | | 0.1845 | G |
| 2616 | 0.0048 | 0.0308 | | 0.1204 | 0.503 | | 0.341 | G |
| 2620 | 0.0047 | 0.0119 | | 0.0051 | 0.9675 | | 0.0107 | G |
| 2630 | 0.0046 | 0.0033 | | 0.0002 | 0.9915 | | 0.0003 | G |
| 2646 | 0.0051 | 0.1002 | | 0.0042 | 0.8876 | | 0.0029 | G |
| 2687 | 0.0046 | 0.9913 | | 0.0017 | 0.0021 | | 0.0002 | A |
| 2714 | 0.0046 | 0.0008 | | 0.1272 | 0.0004 | | 0.867 | T |
| 2736 | 0.0047 | 0.9938 | | 0.0002 | 0.0011 | | 0.0002 | A |
| 2747 | 0.0044 | 0.8466 | | 0.0116 | 0.136 | | 0.0013 | A |
| 2756 | 0.0044 | 0.0007 | | 0.87 | 0.1161 | | 0.0087 | C |
| 2770 | 0.0043 | 0.1912 | | 0.3092 | 0.0916 | | 0.4036 | T |
| 2779 | 0.0042 | 0.1723 | | 0.2773 | 0.1521 | | 0.3941 | T |
| 2788 | 0.0042 | 0.089 | | 0.403 | 0.1041 | | 0.3997 | C |
| 2803 | 0.0041 | 0.0805 | | 0.1129 | 0.4521 | | 0.3504 | G |
| 2807 | 0.004 | 0.0761 | | 0.1803 | 0.7215 | | 0.0179 | G |
| 2834 | 0.0039 | 0.0009 | | 0.002 | 0.9919 | | 0.0012 | G |
| 2864 | 0.0037 | 0.9945 | | 0.0001 | 0.0014 | | 0.0002 | A |
| 2885 | 0.0037 | 0.9943 | | 0.0001 | 0.0017 | | 0.0001 | A |
| 2891 | 0.0038 | 0.991 | | 0.003 | 0.002 | | 0.0002 | A |
| 2913 | 0.0037 | 0.0058 | | 0.7202 | 0.1925 | | 0.0778 | C |
| 2918 | 0.0036 | 0.08 | | 0.249 | 0.3837 | | 0.2836 | G |
| 2932 | 0.0035 | 0.2286 | | 0.0788 | 0.5779 | | 0.111 | G |
| 2935 | 0.0034 | 0.1604 | | 0.1421 | 0.5093 | | 0.1847 | G |
| 2943 | 0.0035 | 0.3121 | | 0.0667 | 0.3905 | | 0.227 | G |
| 2952 | 0.0033 | 0.1433 | | 0.0049 | 0.7316 | | 0.1167 | G |
| 2959 | 0.0033 | 0.0139 | | 0.8498 | 0.0275 | | 0.1054 | C |
| 2965 | 0.0033 | 0.0002 | | 0.0055 | 0.0002 | | 0.9908 | T |
| 2972 | 0.0033 | 0.9948 | | 0.0001 | 0.0015 | | 0.0002 | A |
| 2978 | 0.0031 | 0.9954 | | 0.0001 | 0.0011 | | 0.0002 | A |
| 2983 | 0.003 | 0.0134 | | 0.0074 | 0.0065 | | 0.9698 | T |
| 2986 | 0.0028 | 0.9655 | | 0.0084 | 0.0169 | | 0.0063 | A |
| 3010 | 0.0028 | 0.0002 | | 0.9914 | 0.0003 | | 0.0054 | C |
| 3017 | 0.0027 | 0.0084 | | 0.9337 | 0.008 | | 0.0472 | C |
| 3021 | 0.0026 | 0.0201 | | 0.0818 | 0.872 | | 0.0234 | G |
| 3027 | 0.0026 | 0.0374 | | 0.4674 | 0.4447 | | 0.0477 | C |
| 3034 | 0.0025 | 0.9647 | | 0.0002 | 0.0322 | | 0.0004 | A |
| 3040 | 0.0025 | 0.0002 | | 0.0025 | 0.0006 | | 0.9941 | T |
| 3050 | 0.0024 | 0.8773 | | 0.0003 | 0.1191 | | 0.0006 | A |
| 3054 | 0.0025 | 0.5013 | | 0.2011 | 0.1919 | | 0.103 | A |
| 3055 | 0.0142 | 0.0451 | | 0.1648 | 0.4944 | | 0.2811 | G |
| 3056 | 0.2688 | 0.5334 | | 0.1084 | 0.0458 | | 0.0435 | A |
| 3057 | 0.2707 | 0.0532 | | 0.1726 | 0.1884 | | 0.315 | T |
| 3058 | 0.2711 | 0.1101 | | 0.3296 | 0.0615 | | 0.2276 | C |
| 3059 | 0.2713 | 0.2313 | | 0.082 | 0.0889 | | 0.3263 | T |
| 3060 | 0.2717 | 0.1199 | | 0.2347 | 0.0983 | | 0.2752 | T |
| 3061 | 0.2736 | 0.1593 | | 0.1219 | 0.287 | | 0.158 | G |
| 3070 | 0.0043 | 0.4046 | | 0.1579 | 0.266 | | 0.1669 | A |
| 3073 | 0.0043 | 0.0715 | | 0.5352 | 0.0397 | | 0.3491 | C |
| 3078 | 0.0034 | 0.0104 | | 0.6597 | 0.1057 | | 0.2206 | C |
| 3084 | 0.0035 | 0.0506 | | 0.0118 | 0.6304 | | 0.3036 | G |
| 3101 | 0.0031 | 0.1993 | | 0.646 | 0.0704 | | 0.0809 | C |
| 3120 | 0.003 | 0.749 | | 0.1508 | 0.0747 | | 0.0223 | A |
| 3142 | 0.0031 | 0.0735 | | 0.0124 | 0.2258 | | 0.6852 | T |
| 3161 | 0.014 | 0.0159 | | 0.1006 | 0.8535 | | 0.0158 | G |
| 3165 | 0.0125 | 0.2566 | | 0.033 | 0.5633 | | 0.1344 | G |
| 3168 | 0.0031 | 0.1114 | | 0.1716 | 0.2154 | | 0.4981 | T |
| 3169 | 0.2673 | 0.1744 | | 0.0834 | 0.1886 | | 0.2861 | T |
| 3175 | 0.277 | 0.1418 | | 0.0134 | 0.2879 | | 0.28 | G |
| 3176 | 0.3564 | 0.0096 | | 0.0249 | 0.2002 | | 0.4088 | T |
| 3183 | 0.0019 | 0.1205 | | 0.1109 | 0.6731 | | 0.0935 | G |
| 3188 | 0.0018 | 0.9948 | | 0.0002 | 0.0029 | | 0.0002 | A |
| 3191 | 0.0018 | 0.994 | | 0.0001 | 0.0022 | | 0.0018 | A |
| 3194 | 0.0019 | 0.9956 | | 0.0002 | 0.0019 | | 0.0004 | A |
| 3198 | 0.0017 | 0.065 | | 0.0066 | 0.9191 | | 0.0077 | G |
| 3199 | 0.0015 | 0.3413 | | 0.2335 | 0.2335 | | 0.1901 | A |
| 3200 | 0.0015 | 0.1458 | | 0.1558 | 0.1575 | | 0.5392 | T |
| 3201 | 0.0017 | 0.034 | | 0.098 | 0.537 | | 0.3292 | G |
| 3202 | 0.0064 | 0.0244 | | 0.2597 | 0.4343 | | 0.275 | G |
| 3203 | 0.0543 | 0.231 | | 0.123 | 0.3432 | | 0.2485 | G |
| 3876 | 0.0015 | 0.0616 | | 0.1192 | 0.2948 | | 0.5227 | T |
| 3877 | 0.0016 | 0.0068 | | 0.8315 | 0.0065 | | 0.1535 | C |
| 3881 | 0.0014 | 0.2764 | | 0.0004 | 0.7213 | | 0.0005 | G |
| 3887 | 0.0014 | 0.0055 | | 0.6189 | 0.3569 | | 0.0172 | C |
| 3891 | 0.0014 | 0.0409 | | 0.1178 | 0.0481 | | 0.7918 | T |
| 3899 | 0.0013 | 0.3545 | | 0.066 | 0.2081 | | 0.37 | T |
| 3906 | 0.0013 | 0.3813 | | 0.1188 | 0.1443 | | 0.3542 | A |
| 3909 | 0.0012 | 0.269 | | 0.1441 | 0.2502 | | 0.3353 | T |
| 3914 | 0.0012 | 0.4938 | | 0.0253 | 0.4628 | | 0.0169 | A |
| 3919 | 0.0011 | 0.0042 | | 0.0004 | 0.9938 | | 0.0003 | G |
| 3923 | 0.0011 | 0.9867 | | 0.0011 | 0.0104 | | 0.0007 | A |
| 3927 | 0.0011 | 0.0043 | | 0.0052 | 0.1216 | | 0.8678 | T |
| 3938 | 0.001 | 0.0266 | | 0.0901 | 0.8623 | | 0.0199 | G |
| 3945 | 0.0011 | 0.2616 | | 0.0011 | 0.7083 | | 0.0278 | G |
| 3952 | 0.0011 | 0.3724 | | 0.0454 | 0.5687 | | 0.0123 | G |
| 3955 | 0.001 | 0.0075 | | 0.8087 | 0.0406 | | 0.1421 | C |
| 3958 | 0.001 | 0.0774 | | 0.6624 | 0.0054 | | 0.2538 | C |
| 3965 | 0.001 | 0.0019 | | 0.6176 | 0.0743 | | 0.3052 | C |
| 3977 | 0.001 | 0.2047 | | 0.0003 | 0.7929 | | 0.001 | G |
| 3984 | 0.001 | 0.0419 | | 0.8055 | 0.0542 | | 0.0973 | C |
| 3994 | 0.001 | 0.0726 | | 0.001 | 0.9238 | | 0.0015 | G |
| 4002 | 0.0009 | 0.0031 | | 0.1742 | 0.3225 | | 0.4991 | T |
| 4011 | 0.0011 | 0.0392 | | 0.677 | 0.0613 | | 0.2214 | C |
| 4015 | 0.001 | 0.0877 | | 0.2623 | 0.4421 | | 0.2068 | G |
| 4019 | 0.0009 | 0.003 | | 0.2455 | 0.4115 | | 0.3389 | G |
| 4027 | 0.0008 | 0.9973 | | 0.0001 | 0.0017 | | 0.0001 | A |
| 4037 | 0.0009 | 0.0001 | | 0.0013 | 0.0001 | | 0.9975 | T |
| 4041 | 0.0008 | 0.0001 | | 0.2151 | 0 | | 0.784 | T |
| 4049 | 0.0008 | 0.9977 | | 0.0001 | 0.0011 | | 0.0002 | A |
| 4056 | 0.0008 | 0.0034 | | 0.0008 | 0.9938 | | 0.001 | G |
| 4063 | 0.0008 | 0.0219 | | 0.8584 | 0.0776 | | 0.0412 | C |
| 4067 | 0.0007 | 0.0003 | | 0.0208 | 0.0001 | | 0.9781 | T |
| 4073 | 0.0007 | 0.7129 | | 0.0016 | 0.0287 | | 0.2558 | A |
| 4085 | 0.0008 | 0.0004 | | 0.0001 | 0.9984 | | 0.0002 | G |
| 4089 | 0.0006 | 0.0128 | | 0.0017 | 0.0004 | | 0.9845 | T |
| 4094 | 0.0006 | 0.046 | | 0.0082 | 0.0002 | | 0.945 | T |
| 4105 | 0.0006 | 0.0004 | | 0 | 0.9988 | | 0.0001 | G |
| 4110 | 0.0008 | 0.0004 | | 0 | 0.9986 | | 0.0001 | G |
| 4116 | 0.0008 | 0.0137 | | 0.0999 | 0.0035 | | 0.882 | T |
| 4121 | 0.0008 | 0.2278 | | 0.0001 | 0.7697 | | 0.0015 | G |
| 4128 | 0.0008 | 0.4437 | | 0.0008 | 0.5475 | | 0.0069 | G |
| 4132 | 0.0008 | 0.0033 | | 0.0001 | 0.9911 | | 0.0047 | G |
| 4141 | 0.0008 | 0.0263 | | 0 | 0.9723 | | 0.0005 | G |
| 4166 | 0.0006 | 0.0008 | | 0.0107 | 0.0005 | | 0.9875 | T |
| 4170 | 0.0006 | 0.9773 | | 0.0006 | 0.0209 | | 0.0005 | A |
| 4177 | 0.0006 | 0.931 | | 0.0001 | 0.0681 | | 0.0002 | A |
| 4183 | 0.0006 | 0.1846 | | 0.4958 | 0.0145 | | 0.3042 | C |
| 4195 | 0.0005 | 0.0611 | | 0.0003 | 0.9357 | | 0.0024 | G |
| 4198 | 0.0006 | 0.0006 | | 0.0001 | 0.9985 | | 0.0003 | G |
| 4213 | 0.0007 | 0.0047 | | 0.993 | 0.0001 | | 0.0014 | C |
| 4220 | 0.0008 | 0.0032 | | 0.4434 | 0.0042 | | 0.5483 | T |
| 4225 | 0.0008 | 0.0003 | | 0.4541 | 0.0014 | | 0.5432 | T |
| 4229 | 0.0007 | 0.9953 | | 0.0023 | 0.0015 | | 0.0002 | A |
| 4234 | 0.0007 | 0.0002 | | 0.9986 | 0.0001 | | 0.0004 | C |
| 4242 | 0.0005 | 0.0002 | | 0.9987 | 0 | | 0.0005 | C |
| 4246 | 0.0005 | 0.9446 | | 0.0003 | 0.0084 | | 0.0461 | A |
| 4250 | 0.0005 | 0.9842 | | 0.0002 | 0.0022 | | 0.0128 | A |
| 4268 | 0.0005 | 0.0202 | | 0.0002 | 0.9785 | | 0.0005 | G |
| 4272 | 0.0004 | 0.035 | | 0.0777 | 0.8649 | | 0.0219 | G |
| 4278 | 0.0004 | 0.0001 | | 0.9954 | 0.0003 | | 0.0037 | C |
| 4282 | 0.0006 | 0.2271 | | 0.0145 | 0.6208 | | 0.1367 | G |
| 4288 | 0.0005 | 0.8068 | | 0.002 | 0.0778 | | 0.1127 | A |
| 4293 | 0.0008 | 0.0115 | | 0.8254 | 0.0005 | | 0.1617 | C |
| 4301 | 0.0008 | 0.0033 | | 0.0005 | 0.9937 | | 0.0014 | G |
| 4304 | 0.0004 | 0.9984 | | 0 | 0.0011 | | 0.0001 | A |
| 4313 | 0.0004 | 0.0001 | | 0.2174 | 0.0001 | | 0.782 | T |
| 4326 | 0.0004 | 0.1291 | | 0.4121 | 0.4552 | | 0.0032 | G |
| 4329 | 0.0004 | 0.15 | | 0.4116 | 0.3193 | | 0.1187 | C |
| 4333 | 0.0004 | 0.2211 | | 0.0614 | 0.6782 | | 0.039 | G |
| 4343 | 0.0003 | 0.0001 | | 0.0014 | 0.0001 | | 0.998 | T |
| 4349 | 0.0003 | 0.9979 | | 0.0007 | 0.0008 | | 0.0002 | A |
| 4357 | 0.0004 | 0.0358 | | 0.003 | 0.9531 | | 0.0074 | G |
| 4361 | 0.0004 | 0.0003 | | 0.9009 | 0.0879 | | 0.0104 | C |
| 4366 | 0.0004 | 0.0009 | | 0.6119 | 0.1216 | | 0.2651 | C |
| 4370 | 0.0003 | 0.0011 | | 0.0003 | 0.9981 | | 0.0001 | G |
| 4375 | 0.0003 | 0.3276 | | 0.0029 | 0.61 | | 0.0591 | G |
| 4380 | 0.0003 | 0.0496 | | 0.5616 | 0.0178 | | 0.3707 | C |
| 4383 | 0.0003 | 0.0001 | | 0.9303 | 0.0099 | | 0.0592 | C |
| 4388 | 0.0003 | 0.0002 | | 0.0037 | 0.0001 | | 0.9956 | T |
| 4401 | 0.0003 | 0.0042 | | 0.0002 | 0.9894 | | 0.0059 | G |
| 4409 | 0.0003 | 0.9975 | | 0.0004 | 0.0015 | | 0.0003 | A |
| 4419 | 0.0003 | 0.0015 | | 0.0005 | 0.9972 | | 0.0004 | G |
| 4423 | 0.0003 | 0.997 | | 0.0005 | 0.0018 | | 0.0003 | A |
| 4437 | 0.0003 | 0.0009 | | 0.0001 | 0.9986 | | 0.0001 | G |
| 4441 | 0.0002 | 0.0261 | | 0.0099 | 0.9636 | | 0.0001 | G |
| 4445 | 0.0003 | 0.3696 | | 0.0167 | 0.5629 | | 0.0505 | G |
| 4449 | 0.0002 | 0.055 | | 0.1251 | 0.003 | | 0.8166 | T |
| 4453 | 0.0002 | 0.0095 | | 0.0004 | 0.9848 | | 0.005 | G |
| 4457 | 0.0002 | 0.8279 | | 0.0031 | 0.1308 | | 0.0379 | A |
| 4465 | 0.0003 | 0.1586 | | 0.2486 | 0.0026 | | 0.5899 | T |
| 4472 | 0.0002 | 0.0001 | | 0.9988 | 0.0001 | | 0.0008 | C |
| 4476 | 0.0002 | 0.2651 | | 0.1217 | 0.6119 | | 0.001 | G |
| 4487 | 0.0003 | 0.0014 | | 0.0879 | 0.9097 | | 0.0005 | G |
| 4492 | 0.0002 | 0.0068 | | 0.9474 | 0.0027 | | 0.0428 | C |
| 4531 | 0.0003 | 0.0062 | | 0.9919 | 0.0011 | | 0.0005 | C |
| 4534 | 0.0002 | 0.9912 | | 0.0022 | 0.003 | | 0.0032 | A |
| 4542 | 0.0002 | 0.0001 | | 0.9937 | 0.0056 | | 0.0004 | C |
| 4604 | 0.0002 | 0.9974 | | 0.0003 | 0.0016 | | 0.0004 | A |
| 4608 | 0.0002 | 0.0117 | | 0.6457 | 0.0032 | | 0.3392 | C |
| 4613 | 0.0002 | 0.0004 | | 0.0136 | 0.0138 | | 0.972 | T |
| 4617 | 0.0002 | 0.0005 | | 0.0001 | 0.9991 | | 0.0001 | G |
| 4621 | 0.0002 | 0.0022 | | 0.0002 | 0.9972 | | 0.0002 | G |
| 4628 | 0.0003 | 0.308 | | 0.014 | 0.6346 | | 0.0431 | G |
| 4632 | 0.0002 | 0.9979 | | 0.0001 | 0.0016 | | 0.0002 | A |
| 4637 | 0.0002 | 0.0001 | | 0.997 | 0.0002 | | 0.0025 | C |
| 4646 | 0.0002 | 0.0002 | | 0.0013 | 0.0001 | | 0.9982 | T |
| 4653 | 0.0002 | 0.0003 | | 0 | 0.9991 | | 0.0003 | G |
| 4656 | 0.0002 | 0.9938 | | 0.0012 | 0.0045 | | 0.0003 | A |
| 4662 | 0.0002 | 0.0105 | | 0.0028 | 0.9855 | | 0.0009 | G |
| 4667 | 0.0002 | 0.9974 | | 0.0001 | 0.0017 | | 0.0006 | A |
| 4674 | 0.0012 | 0.0037 | | 0.8372 | 0.0017 | | 0.1561 | C |
| 4684 | 0.0002 | 0.9978 | | 0.0002 | 0.0016 | | 0.0002 | A |
| 4689 | 0.0002 | 0.0003 | | 0.9985 | 0.0004 | | 0.0007 | C |
| 4702 | 0.0002 | 0.0009 | | 0.0005 | 0.9908 | | 0.0076 | G |
| 4722 | 0.0002 | 0.0006 | | 0.0001 | 0.999 | | 0.0001 | G |
| 4730 | 0.0004 | 0.0442 | | 0.634 | 0.0142 | | 0.307 | C |
| 4734 | 0.0002 | 0.0001 | | 0.9967 | 0.0002 | | 0.0028 | C |
| 4740 | 0.0001 | 0.0001 | | 0.9988 | 0.0001 | | 0.0006 | C |
| 4746 | 0.0002 | 0.9709 | | 0.0091 | 0.0144 | | 0.0051 | A |
| 4752 | 0.0002 | 0.1288 | | 0.0056 | 0.8521 | | 0.0129 | G |
| 4775 | 0.0001 | 0.9979 | | 0.0001 | 0.0014 | | 0.0003 | A |
| 4782 | 0.0001 | 0.0001 | | 0.9988 | 0.0001 | | 0.0008 | C |
| 4786 | 0.0002 | 0.0093 | | 0.0037 | 0.0003 | | 0.9866 | T |
| 4789 | 0.0008 | 0.0002 | | 0.995 | 0.0002 | | 0.0038 | C |
| 4795 | 0.0002 | 0.0004 | | 0.9978 | 0.0001 | | 0.0013 | C |
| 4804 | 0.0002 | 0.0002 | | 0.0012 | 0.0001 | | 0.9983 | T |
| 4815 | 0.0001 | 0.9923 | | 0.0004 | 0.0069 | | 0.0003 | A |
| 4821 | 0.0001 | 0.0002 | | 0.9988 | 0.0001 | | 0.0007 | C |
| 4827 | 0.0002 | 0.0009 | | 0.0004 | 0.998 | | 0.0003 | G |
| 4882 | 0.0002 | 0.0004 | | 0 | 0.9992 | | 0.0001 | G |
| 4888 | 0.0001 | 0.0034 | | 0.0001 | 0.996 | | 0.0003 | G |
| 4895 | 0.0002 | 0.9854 | | 0.0002 | 0.0048 | | 0.0094 | A |
| 4898 | 0.0001 | 0.0008 | | 0.0001 | 0.9989 | | 0.0001 | G |
| 4904 | 0.0002 | 0.0013 | | 0.0002 | 0.9982 | | 0.0001 | G |
| 4908 | 0.0001 | 0.0001 | | 0.9991 | 0.0002 | | 0.0005 | C |
| 4915 | 0.0002 | 0.9884 | | 0.0002 | 0.0015 | | 0.0097 | A |
| 4937 | 0.0001 | 0.0008 | | 0.0004 | 0.9982 | | 0.0003 | G |
| 4941 | 0.0001 | 0.0001 | | 0.9992 | 0.0001 | | 0.0005 | C |
| 4947 | 0.0001 | 0.998 | | 0.0002 | 0.0013 | | 0.0003 | A |
| 4956 | 0.0002 | 0.0014 | | 0.0005 | 0.9974 | | 0.0004 | G |
| 4960 | 0.0001 | 0.0003 | | 0.0087 | 0.0006 | | 0.9902 | T |
| 4965 | 0.0001 | 0.3067 | | 0.0173 | 0.6675 | | 0.0082 | G |
| 4972 | 0.0006 | 0.1061 | | 0.0006 | 0.8913 | | 0.0014 | G |
| 4990 | 0.0002 | 0.0155 | | 0.0017 | 0.9825 | | 0.0002 | G |
| 4994 | 0.0001 | 0.0005 | | 0.0001 | 0.9991 | | 0.0001 | G |
| 5003 | 0.0002 | 0.9809 | | 0 | 0.0187 | | 0.0002 | A |
| 5012 | 0.0001 | 0.9976 | | 0.0001 | 0.0021 | | 0.0001 | A |
| 5020 | 0.0001 | 0.0003 | | 0.001 | 0.0005 | | 0.998 | T |
| 5030 | 0.0002 | 0.553 | | 0.3311 | 0.0004 | | 0.1152 | A |
| 5033 | 0.0001 | 0.0043 | | 0.0016 | 0.0034 | | 0.9906 | T |
| 5042 | 0.0002 | 0.0001 | | 0.0008 | 0.0001 | | 0.9987 | T |
| 5045 | 0.0001 | 0.0092 | | 0.3132 | 0.6665 | | 0.011 | G |
| 5050 | 0.0001 | 0.0005 | | 0.6181 | 0.379 | | 0.0022 | C |
| 5054 | 0.0001 | 0.5607 | | 0.0013 | 0.3601 | | 0.0777 | A |
| 5060 | 0.0004 | 0.0001 | | 0.9987 | 0.0001 | | 0.0006 | C |
| 5098 | 0.0003 | 0.9976 | | 0.0004 | 0.0013 | | 0.0002 | A |
| 5103 | 0.0001 | 0.998 | | 0.0002 | 0.0014 | | 0.0002 | A |
| 5106 | 0.0001 | 0.0001 | | 0.0007 | 0.0002 | | 0.9988 | T |
| 5117 | 0.0003 | 0.0007 | | 0.0002 | 0.9987 | | 0.0001 | G |
| 5123 | 0.0001 | 0.0027 | | 0.0031 | 0.9937 | | 0.0003 | G |
| 5128 | 0.0001 | 0.1584 | | 0.0074 | 0.83 | | 0.004 | G |
| 5135 | 0.0002 | 0.0014 | | 0.7582 | 0.2189 | | 0.0213 | C |
| 5148 | 0.0004 | 0.0025 | | 0.001 | 0.9948 | | 0.0013 | G |
| 5153 | 0.0002 | 0.5287 | | 0.2984 | 0.1704 | | 0.0021 | A |
| 5157 | 0.0002 | 0.9582 | | 0.0009 | 0.038 | | 0.0026 | A |
| 5172 | 0.0004 | 0.9918 | | 0.0013 | 0.0062 | | 0.0003 | A |
| 5247 | 0.0005 | 0.0059 | | 0.2187 | 0.7732 | | 0.0015 | G |
| 5254 | 0.0012 | 0.004 | | 0.8288 | 0.0074 | | 0.1585 | C |
| 5257 | 0.0002 | 0.0002 | | 0.9903 | 0.0033 | | 0.0059 | C |
| 5260 | 0.0001 | 0.0005 | | 0.0017 | 0.0003 | | 0.9973 | T |
| 5264 | 0.0001 | 0.0004 | | 0.0001 | 0.9989 | | 0.0003 | G |
| 5268 | 0.0001 | 0.9983 | | 0.0001 | 0.0013 | | 0.0002 | A |
| 5301 | 0.0002 | 0.0775 | | 0.3602 | 0.0011 | | 0.5609 | T |
| 5305 | 0.0002 | 0.0022 | | 0.3796 | 0.6173 | | 0.0006 | G |
| 5309 | 0.0001 | 0.006 | | 0.6665 | 0.3181 | | 0.0092 | C |
| 5322 | 0.0001 | 0.9901 | | 0.0034 | 0.0016 | | 0.0047 | A |
| 5330 | 0.0003 | 0.0021 | | 0.0017 | 0.9946 | | 0.0011 | G |
| 5336 | 0.0001 | 0.0001 | | 0.9958 | 0.0003 | | 0.0036 | C |
| 5341 | 0.0001 | 0.4895 | | 0.2685 | 0.2397 | | 0.0021 | A |
| 5351 | 0.0004 | 0.9976 | | 0.0003 | 0.0013 | | 0.0003 | A |
| 5360 | 0.0002 | 0.0457 | | 0.6349 | 0.0011 | | 0.3181 | C |
| 5366 | 0.0002 | 0.0253 | | 0.0169 | 0.9469 | | 0.0106 | G |
| 5369 | 0.0001 | 0.0001 | | 0.9513 | 0.0001 | | 0.0484 | C |
| 5377 | 0.0002 | 0.0326 | | 0.9604 | 0.0001 | | 0.0066 | C |
| 5383 | 0.0001 | 0.0023 | | 0.0006 | 0.9964 | | 0.0005 | G |
| 5387 | 0.0001 | 0.0005 | | 0.9908 | 0.0001 | | 0.0084 | C |
| 5392 | 0.0003 | 0.0017 | | 0.0001 | 0.9972 | | 0.0007 | G |
| 5399 | 0.0003 | 0.0002 | | 0.0014 | 0.0002 | | 0.9979 | T |
| 5407 | 0.0001 | 0.0014 | | 0 | 0.9984 | | 0.0001 | G |
| 5410 | 0.0002 | 0.5709 | | 0.1453 | 0.1327 | | 0.1509 | A |
| 5414 | 0.0002 | 0.2297 | | 0.0104 | 0.7522 | | 0.0074 | G |
| 5418 | 0.0002 | 0.0007 | | 0.0525 | 0.404 | | 0.5425 | T |
| 5425 | 0.0003 | 0.0006 | | 0.0001 | 0.994 | | 0.005 | G |
| 5433 | 0.0004 | 0.996 | | 0.0005 | 0.0016 | | 0.0014 | A |
| 5443 | 0.0049 | 0.4214 | | 0.0295 | 0.0136 | | 0.5306 | T |
| 5458 | 0.0005 | 0.0009 | | 0.0003 | 0.9949 | | 0.0034 | G |
| 5467 | 0.0001 | 0.9915 | | 0.0001 | 0.008 | | 0.0003 | A |
| 5486 | 0.0002 | 0.7435 | | 0.2544 | 0.0016 | | 0.0003 | A |
| 5489 | 0.0003 | 0.0079 | | 0.0009 | 0.9564 | | 0.0343 | G |
| 5494 | 0.0002 | 0.003 | | 0.0148 | 0.9215 | | 0.0604 | G |
| 5497 | 0.0001 | 0.0704 | | 0.5744 | 0.0358 | | 0.3193 | C |
| 5503 | 0.0001 | 0.0071 | | 0.7513 | 0.0027 | | 0.2386 | C |
| 5514 | 0.0007 | 0.001 | | 0.0598 | 0.0035 | | 0.935 | T |
| 5548 | 0.0002 | 0.0757 | | 0.0051 | 0.0125 | | 0.9065 | T |
| 5551 | 0.0001 | 0.0812 | | 0.814 | 0.0005 | | 0.104 | C |
| 5564 | 0.0002 | 0.0044 | | 0.0014 | 0.9667 | | 0.0273 | G |
| 5577 | 0.0002 | 0.0078 | | 0.0027 | 0.982 | | 0.0072 | G |
| 5581 | 0.0002 | 0.3181 | | 0.0352 | 0.5758 | | 0.0707 | G |
| 5585 | 0.0001 | 0.0602 | | 0.007 | 0.0152 | | 0.9175 | T |
| 5589 | 0.0002 | 0.0003 | | 0.3391 | 0.0001 | | 0.6602 | T |
| 5598 | 0.0001 | 0.0009 | | 0.0003 | 0.998 | | 0.0007 | G |
| 5603 | 0.0001 | 0.0002 | | 0.0014 | 0.0003 | | 0.9978 | T |
| 5608 | 0.0004 | 0.9945 | | 0.0005 | 0.004 | | 0.0003 | A |
| 5615 | 0.0002 | 0.9974 | | 0.0004 | 0.0015 | | 0.0003 | A |
| 5620 | 0.0001 | 0.9974 | | 0.0002 | 0.002 | | 0.0002 | A |
| 5634 | 0.0003 | 0.1909 | | 0.4046 | 0.4035 | | 0.0005 | C |
| 5640 | 0.0001 | 0.0074 | | 0.689 | 0.0105 | | 0.2929 | C |
| 5644 | 0.0001 | 0.1508 | | 0.1245 | 0.1454 | | 0.5791 | T |
| 5647 | 0.0001 | 0.0003 | | 0.9966 | 0.0001 | | 0.0028 | C |
| 5652 | 0.0002 | 0.0013 | | 0.0017 | 0.0023 | | 0.9944 | T |
| 5656 | 0.0016 | 0.0512 | | 0.0014 | 0.3669 | | 0.5787 | T |
| 5661 | 0.0002 | 0.0027 | | 0.0012 | 0.0019 | | 0.9939 | T |
| 5673 | 0.0002 | 0.0601 | | 0.4036 | 0.0046 | | 0.5313 | T |
| 5678 | 0.0002 | 0.5046 | | 0.0339 | 0.4469 | | 0.0142 | A |
| 5682 | 0.0003 | 0.0441 | | 0.1707 | 0.2756 | | 0.5092 | T |
| 5685 | 0.0002 | 0.1915 | | 0.3843 | 0.0483 | | 0.3755 | C |
| 5690 | 0.0002 | 0.5776 | | 0.1013 | 0.1609 | | 0.1599 | A |
| 5694 | 0.0003 | 0.1059 | | 0.0098 | 0.8727 | | 0.0112 | G |
| 5697 | 0.0004 | 0.1024 | | 0.0203 | 0.7608 | | 0.1161 | G |
| 5701 | 0.0008 | 0.0486 | | 0.0077 | 0.941 | | 0.0017 | G |
| 5705 | 0.0005 | 0.9703 | | 0.0002 | 0.0288 | | 0.0002 | A |
| 5710 | 0.0004 | 0.6213 | | 0.2335 | 0.1272 | | 0.0176 | A |
| 5711 | 0.0002 | 0.0107 | | 0.0181 | 0.9097 | | 0.0613 | G |
| 5714 | 0.0001 | 0.9958 | | 0.0025 | 0.0014 | | 0.0002 | A |
| 5715 | 0.0002 | 0.8657 | | 0.0018 | 0.0296 | | 0.1027 | A |
| 5716 | 0.0002 | 0.3624 | | 0.2752 | 0.3155 | | 0.0466 | A |
| 5717 | 0.0003 | 0.4641 | | 0.2392 | 0.2487 | | 0.0475 | A |
| 5718 | 0.1453 | 0.2631 | | 0.1133 | 0.3012 | | 0.1768 | G |
| 5719 | 0.1644 | 0.2649 | | 0.1498 | 0.2024 | | 0.2182 | A |
| 5720 | 0.1838 | 0.1519 | | 0.1254 | 0.1447 | | 0.3939 | T |
| 5721 | 0.2073 | 0.1881 | | 0.081 | 0.3344 | | 0.189 | G |
| 5722 | 0.2075 | 0.0703 | | 0.1246 | 0.2301 | | 0.3672 | T |
| 5723 | 0.3244 | 0.0804 | | 0.0897 | 0.3547 | | 0.1505 | G |
| 5729 | 0.3812 | 0.4672 | | 0.0088 | 0.0771 | | 0.0656 | A |
| 6901 | 0.0001 | 0.0253 | | 0.0057 | 0.0239 | | 0.9448 | T |
| 6902 | 0.0001 | 0.0003 | | 0.0001 | 0.9993 | | 0.0001 | G |
| 6904 | 0.0002 | 0.9577 | | 0.0269 | 0.0016 | | 0.0136 | A |
| 6910 | 0.0002 | 0.0593 | | 0.9108 | 0.0155 | | 0.0141 | C |
| 6915 | 0.0007 | 0.051 | | 0.0059 | 0.8936 | | 0.0487 | G |
| 6921 | 0.0013 | 0.0015 | | 0.001 | 0.8741 | | 0.1222 | G |
| 6924 | 0.0052 | 0.0029 | | 0.0044 | 0.0015 | | 0.9859 | T |
| 6929 | 0.0006 | 0.9952 | | 0.0004 | 0.0034 | | 0.0003 | A |
| 6935 | 0.0004 | 0.0918 | | 0.7598 | 0.0518 | | 0.0961 | C |
| 6939 | 0.0003 | 0.0073 | | 0.8092 | 0.013 | | 0.1701 | C |
| 6945 | 0.0002 | 0.0798 | | 0.156 | 0.1729 | | 0.591 | T |
| 6949 | 0.0003 | 0.3312 | | 0.0456 | 0.4168 | | 0.2058 | G |
| 6953 | 0.0002 | 0.5655 | | 0.2154 | 0.1751 | | 0.0437 | A |
| 6957 | 0.0002 | 0.4402 | | 0.2208 | 0.0642 | | 0.2745 | A |
| 6964 | 0.003 | 0.0961 | | 0.2678 | 0.5208 | | 0.1122 | G |
| 6968 | 0.003 | 0.9951 | | 0.0003 | 0.0012 | | 0.0004 | A |
| 6981 | 0.002 | 0.6319 | | 0.0004 | 0.3645 | | 0.0012 | A |
| 6993 | 0.0002 | 0.3179 | | 0.0056 | 0.4145 | | 0.2616 | G |
| 7005 | 0.0002 | 0.9926 | | 0.004 | 0.0014 | | 0.0018 | A |
| 7016 | 0.0002 | 0.9978 | | 0.0002 | 0.0016 | | 0.0002 | A |
| 7023 | 0.0002 | 0.0069 | | 0.0004 | 0.992 | | 0.0004 | G |
| 7028 | 0.0002 | 0.0035 | | 0.8307 | 0.1274 | | 0.0382 | C |
| 7032 | 0.0003 | 0.4827 | | 0.3689 | 0.1104 | | 0.0376 | A |
| 7035 | 0.0002 | 0.323 | | 0.6125 | 0.0041 | | 0.0602 | C |
| 7039 | 0.0002 | 0.0005 | | 0.9919 | 0.0033 | | 0.004 | C |
| 7047 | 0.0003 | 0.0011 | | 0.0007 | 0.9966 | | 0.0013 | G |
| 7057 | 0.0001 | 0.001 | | 0.0002 | 0.9985 | | 0.0001 | G |
| 7069 | 0.0002 | 0.0002 | | 0.9983 | 0.0005 | | 0.0008 | C |
| 7075 | 0.0002 | 0.004 | | 0.0177 | 0.0001 | | 0.9779 | T |
| 7087 | 0.0001 | 0.9981 | | 0.0001 | 0.0008 | | 0.0007 | A |
| 7094 | 0.0004 | 0.9982 | | 0.0001 | 0.0011 | | 0.0002 | A |
| 7101 | 0.0002 | 0.0267 | | 0.938 | 0.0035 | | 0.0314 | C |
| 7109 | 0.0001 | 0.0002 | | 0.002 | 0.0001 | | 0.9976 | T |
| 7113 | 0.0002 | 0.6847 | | 0.2602 | 0.0008 | | 0.0541 | A |
| 7117 | 0.0001 | 0.001 | | 0.9336 | 0.0001 | | 0.0651 | C |
| 7122 | 0.0002 | 0.0007 | | 0.0008 | 0.9977 | | 0.0005 | G |
| 7131 | 0.0002 | 0.0002 | | 0.0016 | 0.0001 | | 0.9979 | T |
| 7143 | 0.0002 | 0.0005 | | 0.0001 | 0.9991 | | 0.0001 | G |
| 7153 | 0.0003 | 0.0001 | | 0.9985 | 0.0005 | | 0.0006 | C |
| 7180 | 0.0002 | 0.0001 | | 0.9992 | 0 | | 0.0005 | C |
| 7188 | 0.0002 | 0.9972 | | 0.0007 | 0.0013 | | 0.0005 | A |
| 7194 | 0.0002 | 0.0018 | | 0.0014 | 0.9953 | | 0.0011 | G |
| 7200 | 0.0002 | 0.0004 | | 0.9989 | 0.0001 | | 0.0005 | C |
| 7211 | 0.0002 | 0.9977 | | 0.0005 | 0.0012 | | 0.0003 | A |
| 7215 | 0.0002 | 0.001 | | 0.0008 | 0.9971 | | 0.0006 | G |
| 7221 | 0.0002 | 0.0002 | | 0.9983 | 0.0003 | | 0.0011 | C |
| 7229 | 0.0003 | 0.0008 | | 0.9973 | 0.0002 | | 0.0013 | C |
| 7255 | 0.0002 | 0.0006 | | 0.0005 | 0.998 | | 0.0006 | G |
| 7261 | 0.0002 | 0.0001 | | 0.9988 | 0.0002 | | 0.0006 | C |
| 7267 | 0.0004 | 0.0007 | | 0.0004 | 0.9982 | | 0.0002 | G |
| 7271 | 0.0002 | 0.0005 | | 0.0001 | 0.999 | | 0.0002 | G |
| 7277 | 0.0002 | 0.0004 | | 0.0014 | 0.0012 | | 0.9966 | T |
| 7287 | 0.0005 | 0.9358 | | 0.0017 | 0.0606 | | 0.0012 | A |
| 7298 | 0.0001 | 0.9985 | | 0.0001 | 0.001 | | 0.0002 | A |
| 7302 | 0.0002 | 0.0128 | | 0.0073 | 0.0147 | | 0.9649 | T |
| 7310 | 0.0002 | 0.9985 | | 0.0001 | 0.0009 | | 0.0002 | A |
| 7326 | 0.0002 | 0.0001 | | 0.9991 | 0.0001 | | 0.0005 | C |
| 7336 | 0.0002 | 0.0636 | | 0.0016 | 0.9325 | | 0.0018 | G |
| 7341 | 0.0002 | 0.0539 | | 0.0008 | 0.2602 | | 0.6848 | T |
| 7345 | 0.0002 | 0.9812 | | 0.0001 | 0.0181 | | 0.0003 | A |
| 7350 | 0.0002 | 0.0355 | | 0.0049 | 0.9291 | | 0.0288 | G |
| 7359 | 0.0006 | 0.003 | | 0.0024 | 0.9925 | | 0.0013 | G |
| 7363 | 0.0001 | 0.0602 | | 0.0046 | 0.6125 | | 0.3225 | G |
| 7368 | 0.0005 | 0.0365 | | 0.1106 | 0.3699 | | 0.4824 | T |
| 7372 | 0.0001 | 0.0222 | | 0.125 | 0.8467 | | 0.0058 | G |
| 7376 | 0.0001 | 0.0003 | | 0.9917 | 0.0011 | | 0.0066 | C |
| 7380 | 0.0005 | 0.5481 | | 0.0025 | 0.39 | | 0.0582 | A |
| 7448 | 0.0001 | 0.9819 | | 0.0002 | 0.0176 | | 0.0002 | A |
| 7453 | 0.0002 | 0.0081 | | 0.0003 | 0.9912 | | 0.0001 | G |
| 7459 | 0.0001 | 0.0001 | | 0.9972 | 0.0001 | | 0.0024 | C |
| 7467 | 0.0001 | 0.0015 | | 0.0003 | 0.9977 | | 0.0002 | G |
| 7472 | 0.0001 | 0.0001 | | 0.0016 | 0.0006 | | 0.9975 | T |
| 7476 | 0.0007 | 0.0055 | | 0.0011 | 0.0002 | | 0.9923 | T |
| 7489 | 0.0002 | 0.5601 | | 0.0002 | 0.4384 | | 0.0009 | A |
| 7495 | 0.0002 | 0.1885 | | 0.03 | 0.0007 | | 0.7806 | T |
| 7498 | 0.0002 | 0.0001 | | 0.7056 | 0.0013 | | 0.2928 | C |
| 7502 | 0.0002 | 0.0001 | | 0.9984 | 0.0004 | | 0.0008 | C |
| 7558 | 0.0001 | 0.0014 | | 0.0025 | 0.9954 | | 0.0004 | G |
| 7592 | 0.0003 | 0.0025 | | 0.0002 | 0.9967 | | 0.0002 | G |
| 7604 | 0.0001 | 0.9958 | | 0.0001 | 0.0025 | | 0.0013 | A |
| 7611 | 0.0007 | 0.6493 | | 0.0015 | 0.005 | | 0.3435 | A |
| 7622 | 0.0001 | 0.0014 | | 0.0009 | 0.0001 | | 0.9973 | T |
| 7658 | 0.0005 | 0.0101 | | 0.0625 | 0.016 | | 0.9108 | T |
| 7662 | 0.0003 | 0.9972 | | 0.0002 | 0.0006 | | 0.0015 | A |
| 7666 | 0.0007 | 0.0011 | | 0.4684 | 0.0001 | | 0.5297 | T |
| 7717 | 0.0001 | 0.0002 | | 0.001 | 0.0006 | | 0.998 | T |
| 7736 | 0.0007 | 0.0012 | | 0 | 0.9977 | | 0.0004 | G |
| 7745 | 0.0001 | 0.0009 | | 0.0005 | 0.9978 | | 0.0004 | G |
| 7749 | 0.0001 | 0.0006 | | 0.0004 | 0.9987 | | 0.0002 | G |
| 7753 | 0.0001 | 0.0002 | | 0.8292 | 0.0006 | | 0.1698 | C |
| 7761 | 0.0003 | 0.0026 | | 0.0006 | 0.9013 | | 0.0951 | G |
| 7780 | 0.0002 | 0.0004 | | 0.0011 | 0.0004 | | 0.9978 | T |
| 7790 | 0.0002 | 0.9973 | | 0.0002 | 0.0007 | | 0.0009 | A |
| 7825 | 0.0004 | 0.9965 | | 0.0012 | 0.0013 | | 0.0006 | A |
| 7924 | 0.0052 | 0.992 | | 0.0002 | 0.0023 | | 0.0002 | A |
| 7929 | 0.0003 | 0.0012 | | 0.0003 | 0.998 | | 0.0002 | G |
| 7941 | 0.0003 | 0.1234 | | 0.4992 | 0.3759 | | 0.0011 | C |
| 7946 | 0.0001 | 0.003 | | 0.0003 | 0.9963 | | 0.0002 | G |
| 7951 | 0.0002 | 0.2106 | | 0.5637 | 0.0087 | | 0.2168 | C |
| 7961 | 0.0002 | 0.0975 | | 0.0371 | 0.6621 | | 0.203 | G |
| 7967 | 0.0001 | 0.0052 | | 0.9171 | 0.0009 | | 0.0767 | C |
| 7984 | 0.0002 | 0.0008 | | 0.0054 | 0.993 | | 0.0006 | G |
| 7988 | 0.0001 | 0.0004 | | 0.3128 | 0.0005 | | 0.6863 | T |
| 7998 | 0.0007 | 0.9972 | | 0.0004 | 0.0013 | | 0.0003 | A |
| 8006 | 0.0081 | 0.0062 | | 0.0029 | 0.9796 | | 0.0013 | G |
| 8010 | 0.0001 | 0.0622 | | 0.0037 | 0.9338 | | 0.0002 | G |
| 8014 | 0.0002 | 0.0005 | | 0.7414 | 0.0007 | | 0.2571 | C |
| 8023 | 0.0004 | 0.0012 | | 0.0115 | 0.9753 | | 0.0115 | G |
| 8033 | 0.0001 | 0.0007 | | 0.0002 | 0.9976 | | 0.0013 | G |
| 8037 | 0.0002 | 0.0997 | | 0.1619 | 0.0606 | | 0.6775 | T |
| 8042 | 0.0003 | 0.0772 | | 0.1448 | 0.0522 | | 0.7254 | T |
| 8047 | 0.0004 | 0.1854 | | 0.1027 | 0.1438 | | 0.5675 | T |
| 8057 | 0.0011 | 0.1857 | | 0.1017 | 0.39 | | 0.3213 | G |
| 8061 | 0.0003 | 0.1577 | | 0.0259 | 0.204 | | 0.6119 | T |
| 8066 | 0.0003 | 0.0241 | | 0.3402 | 0.0022 | | 0.633 | T |
| 8092 | 0.0106 | 0.8009 | | 0.0143 | 0.1626 | | 0.0115 | A |
| 8099 | 0.0001 | 0.8025 | | 0.1841 | 0.0095 | | 0.0036 | A |
| 8109 | 0.0002 | 0.001 | | 0.0004 | 0.9978 | | 0.0004 | G |
| 8112 | 0.0001 | 0.0488 | | 0.0522 | 0.0003 | | 0.8986 | T |
| 8120 | 0.0001 | 0.0186 | | 0.8467 | 0.0409 | | 0.0935 | C |
| 8130 | 0.0002 | 0.2963 | | 0.0254 | 0.2746 | | 0.4034 | T |
| 8137 | 0.0002 | 0.0367 | | 0.0095 | 0.8649 | | 0.0883 | G |
| 8141 | 0.0002 | 0.6373 | | 0.1244 | 0.1103 | | 0.1277 | A |
| 8145 | 0.0002 | 0.1793 | | 0.0528 | 0.1371 | | 0.6305 | T |
| 8149 | 0.0007 | 0.0027 | | 0.0018 | 0.9856 | | 0.0091 | G |
| 8153 | 0.0002 | 0.0003 | | 0.0019 | 0.0008 | | 0.9967 | T |
| 8157 | 0.0002 | 0.0224 | | 0.0131 | 0.883 | | 0.0812 | G |
| 8165 | 0.0081 | 0.9096 | | 0.001 | 0.0118 | | 0.0694 | A |
| 8183 | 0.0001 | 0.9969 | | 0.0007 | 0.0018 | | 0.0004 | A |
| 8244 | 0.0002 | 0.9972 | | 0.0001 | 0.0021 | | 0.0003 | A |
| 8284 | 0.0006 | 0.0578 | | 0.0082 | 0.5198 | | 0.4135 | G |
| 8288 | 0.0004 | 0.0387 | | 0.7267 | 0.1216 | | 0.1125 | C |
| 8292 | 0.0002 | 0.0035 | | 0.8056 | 0.0232 | | 0.1673 | C |
| 8296 | 0.0002 | 0.0612 | | 0.6873 | 0.0233 | | 0.2278 | C |
| 8305 | 0.0003 | 0.2741 | | 0.224 | 0.4115 | | 0.09 | G |
| 8309 | 0.0003 | 0.0498 | | 0.3058 | 0.532 | | 0.112 | G |
| 8313 | 0.0002 | 0.12 | | 0.0301 | 0.8353 | | 0.0143 | G |
| 8318 | 0.0002 | 0.002 | | 0.0005 | 0.9948 | | 0.0025 | G |
| 8329 | 0.0002 | 0.0001 | | 0.9957 | 0.0006 | | 0.0033 | C |
| 8333 | 0.0002 | 0.0004 | | 0.0148 | 0.0001 | | 0.9844 | T |
| 8351 | 0.0003 | 0.0017 | | 0.6089 | 0.0001 | | 0.3888 | C |
| 8363 | 0.0001 | 0.996 | | 0.0014 | 0.001 | | 0.0013 | A |
| 8365 | 0.0018 | 0.991 | | 0.0028 | 0.0019 | | 0.0023 | A |
| 8410 | 0.0004 | 0.0003 | | 0.9935 | 0.0025 | | 0.0033 | C |
| 8416 | 0.0001 | 0.0079 | | 0.7502 | 0.0362 | | 0.2055 | C |
| 8422 | 0.0002 | 0.1031 | | 0.3007 | 0.3161 | | 0.2798 | G |
| 8427 | 0.0005 | 0.0607 | | 0.2428 | 0.2553 | | 0.4406 | T |
| 8432 | 0.0002 | 0.1465 | | 0.0209 | 0.7686 | | 0.0631 | G |
| 8436 | 0.0001 | 0.139 | | 0.0057 | 0.8241 | | 0.0308 | G |
| 8440 | 0.0001 | 0.6136 | | 0.0293 | 0.2554 | | 0.1014 | A |
| 8445 | 0.0065 | 0.5524 | | 0.1752 | 0.2185 | | 0.0473 | A |
| 8449 | 0.0001 | 0.034 | | 0.436 | 0.3604 | | 0.1693 | C |
| 8521 | 0.1047 | 0.012 | | 0.007 | 0.2465 | | 0.6297 | T |
| 8524 | 0.0001 | 0.0038 | | 0.0081 | 0.7954 | | 0.1925 | G |
| 8530 | 0.0008 | 0.0002 | | 0.9831 | 0.0109 | | 0.0049 | C |
| 8533 | 0.0003 | 0.5974 | | 0.134 | 0.0839 | | 0.1843 | A |
| 8537 | 0.0002 | 0.0622 | | 0.047 | 0.1829 | | 0.7077 | T |
| 8543 | 0.0002 | 0.0102 | | 0.1133 | 0.0895 | | 0.7867 | T |
| 8553 | 0.0003 | 0.0136 | | 0.2745 | 0.4175 | | 0.294 | G |
| 8565 | 0.0002 | 0.088 | | 0.0407 | 0.8518 | | 0.0192 | G |
| 8571 | 0.0001 | 0.8704 | | 0.0004 | 0.0801 | | 0.0488 | A |
| 8575 | 0.0001 | 0.5854 | | 0.0005 | 0.0684 | | 0.3455 | A |
| 8578 | 0.0006 | 0.9978 | | 0.0002 | 0.0012 | | 0.0001 | A |
| 8585 | 0.0001 | 0.0001 | | 0.999 | 0.0001 | | 0.0007 | C |
| 8591 | 0.0001 | 0.0008 | | 0.0096 | 0.1863 | | 0.8032 | T |
| 8634 | 0.0001 | 0.0461 | | 0.0051 | 0.9396 | | 0.009 | G |
| 8639 | 0.0002 | 0.0478 | | 0.1394 | 0.5871 | | 0.2254 | G |
| 8643 | 0.0002 | 0.2725 | | 0.3079 | 0.1503 | | 0.2688 | C |
| 8647 | 0.0002 | 0.4841 | | 0.0504 | 0.1504 | | 0.3146 | A |
| 8655 | 0.0002 | 0.4709 | | 0.0274 | 0.4263 | | 0.075 | A |
| 8659 | 0.0002 | 0.5619 | | 0.0217 | 0.278 | | 0.1381 | A |
| 8662 | 0.0001 | 0.0016 | | 0.9974 | 0.0001 | | 0.0007 | C |
| 8676 | 0.0001 | 0.0002 | | 0.0014 | 0.0001 | | 0.9982 | T |
| 8684 | 0.0003 | 0.3075 | | 0.0179 | 0.0589 | | 0.6152 | T |
| 8690 | 0.0001 | 0.0008 | | 0.0003 | 0.9983 | | 0.0003 | G |
| 8693 | 0.0001 | 0.9939 | | 0.0001 | 0.0056 | | 0.0003 | A |
| 8696 | 0.0001 | 0.0458 | | 0.0016 | 0.9512 | | 0.0012 | G |
| 8699 | 0.0002 | 0.0208 | | 0.0042 | 0.0879 | | 0.8869 | T |
| 8704 | 0.0002 | 0.3433 | | 0.074 | 0.5252 | | 0.0572 | G |
| 8708 | 0.0001 | 0.1053 | | 0.6178 | 0.0383 | | 0.2384 | C |
| 8712 | 0.0003 | 0.4017 | | 0.0649 | 0.3538 | | 0.1791 | A |
| 8717 | 0.0002 | 0.0927 | | 0.0249 | 0.8032 | | 0.0789 | G |
| 8721 | 0.0004 | 0.2629 | | 0.1063 | 0.2859 | | 0.3445 | T |
| 8726 | 0.0003 | 0.9256 | | 0.0181 | 0.0112 | | 0.0446 | A |
| 8736 | 0.0003 | 0.0203 | | 0.0011 | 0.9746 | | 0.0033 | G |
| 8749 | 0.0004 | 0.6837 | | 0.0044 | 0.3111 | | 0.0004 | A |
| 8753 | 0.0005 | 0.03 | | 0.0073 | 0.9594 | | 0.0027 | G |
| 8757 | 0.0001 | 0.0202 | | 0.0001 | 0.9792 | | 0.0002 | G |
| 8761 | 0.0005 | 0.507 | | 0.0593 | 0.2023 | | 0.2308 | A |
| 8766 | 0.0002 | 0.4701 | | 0.0208 | 0.427 | | 0.0818 | A |
| 8770 | 0.0002 | 0.6647 | | 0.0116 | 0.298 | | 0.0253 | A |
| 8774 | 0.0002 | 0.1267 | | 0.1391 | 0.7103 | | 0.0235 | G |
| 8778 | 0.0002 | 0.0044 | | 0.1918 | 0.0165 | | 0.7871 | T |
| 9072 | 0.0003 | 0.1215 | | 0.0003 | 0.8777 | | 0.0002 | G |
| 9076 | 0.0002 | 0.0006 | | 0.0002 | 0.9987 | | 0.0002 | G |
| 9084 | 0.0005 | 0.997 | | 0.0001 | 0.0016 | | 0.0007 | A |
| 9089 | 0.0001 | 0.9976 | | 0.0001 | 0.0015 | | 0.0006 | A |
| 9094 | 0.0002 | 0.0004 | | 0.0565 | 0.0001 | | 0.9427 | T |
| 9098 | 0.0005 | 0.0258 | | 0.002 | 0.03 | | 0.9415 | T |
| 9101 | 0.0004 | 0.0243 | | 0.8687 | 0.0165 | | 0.0899 | C |
| 9106 | 0.0001 | 0.0143 | | 0.8906 | 0.0818 | | 0.013 | C |
| 9116 | 0.0002 | 0.4031 | | 0.1096 | 0.0815 | | 0.4054 | T |
| 9120 | 0.0004 | 0.2088 | | 0.1157 | 0.3978 | | 0.277 | G |
| 9127 | 0.0001 | 0.0164 | | 0.0005 | 0.9826 | | 0.0002 | G |
| 9132 | 0.0002 | 0.0072 | | 0.0017 | 0.0006 | | 0.9902 | T |
| 9140 | 0.0001 | 0.0004 | | 0 | 0.9993 | | 0.0001 | G |
| 9148 | 0.0001 | 0.001 | | 0.0009 | 0.0692 | | 0.9286 | T |
| 9165 | 0.0001 | 0.9983 | | 0.0001 | 0.0012 | | 0.0002 | A |
| 9173 | 0.0002 | 0.0034 | | 0.0011 | 0.9946 | | 0.0003 | G |
| 9177 | 0.0001 | 0.0642 | | 0.9119 | 0.015 | | 0.0088 | C |
| 9180 | 0.0001 | 0.084 | | 0.0003 | 0.9154 | | 0.0001 | G |
| 9185 | 0.0006 | 0.0005 | | 0.0002 | 0.9984 | | 0.0002 | G |
| 9206 | 0.0002 | 0.0002 | | 0.0015 | 0.0006 | | 0.9974 | T |
| 9216 | 0.0002 | 0.0143 | | 0.0001 | 0.9848 | | 0.0005 | G |
| 9258 | 0.0001 | 0.8468 | | 0.0002 | 0.1525 | | 0.0002 | A |
| 9265 | 0.0001 | 0.9959 | | 0.0012 | 0.0018 | | 0.0009 | A |
| 9274 | 0.0008 | 0.9971 | | 0.0001 | 0.0017 | | 0.0002 | A |
| 9332 | 0.0002 | 0.0002 | | 0.0009 | 0.0002 | | 0.9984 | T |
| 9342 | 0.0001 | 0.0033 | | 0.0149 | 0.9175 | | 0.0641 | G |
| 9351 | 0.0001 | 0.0001 | | 0.9917 | 0.0005 | | 0.0075 | C |
| 9355 | 0.0002 | 0.0518 | | 0.0005 | 0.9027 | | 0.0448 | G |
| 9359 | 0.0001 | 0.0002 | | 0.3397 | 0.0001 | | 0.6599 | T |
| 9365 | 0.0001 | 0.991 | | 0.0001 | 0.0015 | | 0.0072 | A |
| 9387 | 0.0002 | 0.0048 | | 0.0006 | 0.993 | | 0.0008 | G |
| 9391 | 0.0001 | 0.9985 | | 0.0001 | 0.0012 | | 0.0002 | A |
| 9395 | 0.0001 | 0.0007 | | 0.0005 | 0.3196 | | 0.679 | T |
| 9457 | 0.0002 | 0.9974 | | 0.0007 | 0.0009 | | 0.0007 | A |
| 9465 | 0.0001 | 0.0002 | | 0.0055 | 0.0003 | | 0.9938 | T |
| 9473 | 0.0005 | 0.2723 | | 0.3971 | 0.1204 | | 0.2097 | C |
| 9481 | 0.0003 | 0.4049 | | 0.0814 | 0.1093 | | 0.404 | A |
| 9488 | 0.0001 | 0.0123 | | 0.0821 | 0.8907 | | 0.0146 | G |
| 9492 | 0.0007 | 0.0818 | | 0.0165 | 0.8765 | | 0.0244 | G |
| 9505 | 0.0002 | 0.9411 | | 0.0288 | 0.0029 | | 0.0269 | A |
| 9509 | 0.0002 | 0.1992 | | 0.0006 | 0.7982 | | 0.0018 | G |
| 9521 | 0.0005 | 0.0011 | | 0.0002 | 0.998 | | 0.0001 | G |
| 9530 | 0.0008 | 0.9968 | | 0.0001 | 0.0018 | | 0.0003 | A |
| 9543 | 0.0001 | 0.9972 | | 0.0007 | 0.0017 | | 0.0002 | A |
| 9547 | 0.0001 | 0.0003 | | 0.855 | 0.0027 | | 0.1418 | C |
| 9551 | 0.0002 | 0.9361 | | 0.0077 | 0.014 | | 0.042 | A |
| 9560 | 0.0005 | 0.0002 | | 0.9732 | 0.0001 | | 0.0259 | C |
| 9563 | 0.0001 | 0.0001 | | 0.9953 | 0.0002 | | 0.0041 | C |
| 9564 | 0.0002 | 0.5378 | | 0.013 | 0.44 | | 0.0088 | A |
| 9569 | 0.0001 | 0.3219 | | 0.0003 | 0.6766 | | 0.001 | G |
| 9627 | 0.0002 | 0.0244 | | 0.0115 | 0.0013 | | 0.9625 | T |
| 9717 | 0.0007 | 0.0176 | | 0.0049 | 0.8908 | | 0.086 | G |
| 9723 | 0.0002 | 0.0022 | | 0.0004 | 0.9968 | | 0.0002 | G |
| 9726 | 0.0002 | 0.0015 | | 0.9893 | 0.0039 | | 0.0049 | C |
| 9742 | 0.0002 | 0.0008 | | 0.0004 | 0.9981 | | 0.0004 | G |
| 9747 | 0.0004 | 0.9894 | | 0.0015 | 0.0025 | | 0.0061 | A |
| 9786 | 0.0001 | 0.9975 | | 0.0002 | 0.0017 | | 0.0002 | A |
| 9792 | 0.0021 | 0.0829 | | 0.0007 | 0.9123 | | 0.0004 | G |
| 9815 | 0.0002 | 0.0046 | | 0.0032 | 0.9902 | | 0.0015 | G |
| 9818 | 0.0002 | 0.0001 | | 0.9965 | 0.0007 | | 0.0023 | C |
| 9883 | 0.0003 | 0.3173 | | 0.0004 | 0.6813 | | 0.0004 | G |
| 9888 | 0.0002 | 0.2912 | | 0.0161 | 0.6862 | | 0.0061 | G |
| 9894 | 0.0003 | 0.0219 | | 0.7098 | 0.1407 | | 0.1271 | C |
| 9898 | 0.0003 | 0.0242 | | 0.2368 | 0.0122 | | 0.7262 | T |
| 9902 | 0.0003 | 0.0714 | | 0.4266 | 0.0309 | | 0.4706 | T |
| 9907 | 0.001 | 0.2251 | | 0.1987 | 0.0647 | | 0.5104 | T |
| 9911 | 0.0002 | 0.0002 | | 0.9785 | 0.0003 | | 0.0205 | C |
| 9922 | 0.0002 | 0.0025 | | 0.0278 | 0.0073 | | 0.9621 | T |
| 9928 | 0.0005 | 0.0637 | | 0.0001 | 0.9347 | | 0.0008 | G |
| 10000 | 0.0001 | 0.0423 | | 0.006 | 0.946 | | 0.0054 | G |
| 10009 | 0.0002 | 0.264 | | 0.138 | 0.3592 | | 0.2385 | G |
| 10013 | 0.0002 | 0.0797 | | 0.7856 | 0.0334 | | 0.1008 | C |
| 10020 | 0.0002 | 0.1768 | | 0.2826 | 0.0663 | | 0.4739 | T |
| 10028 | 0.0002 | 0.2339 | | 0.0362 | 0.5819 | | 0.1476 | G |
| 10032 | 0.002 | 0.2313 | | 0.0857 | 0.0811 | | 0.5997 | T |
| 10058 | 0.0002 | 0.5019 | | 0.0763 | 0.0655 | | 0.3559 | A |
| 10062 | 0.0002 | 0.8942 | | 0.0739 | 0.007 | | 0.0246 | A |
| 10066 | 0.0003 | 0.0011 | | 0.9516 | 0.0013 | | 0.0455 | C |
| 10071 | 0.0002 | 0.0011 | | 0.0049 | 0.0004 | | 0.9933 | T |
| 10078 | 0.0002 | 0.001 | | 0 | 0.9987 | | 0.0001 | G |
| 10082 | 0.0002 | 0.998 | | 0.0001 | 0.0015 | | 0.0003 | A |
| 10088 | 0.0002 | 0.0001 | | 0.9992 | 0.0001 | | 0.0004 | C |
| 10149 | 0.0002 | 0.1289 | | 0.0008 | 0.8694 | | 0.0005 | G |
| 10153 | 0.0004 | 0.0003 | | 0.9313 | 0.0036 | | 0.0641 | C |
| 10157 | 0.0001 | 0.0002 | | 0.0014 | 0.0003 | | 0.9979 | T |
| 10254 | 0.0001 | 0.0095 | | 0.1372 | 0.85 | | 0.003 | G |
| 10257 | 0.0001 | 0.9971 | | 0.0004 | 0.0017 | | 0.0006 | A |
| 10264 | 0.0013 | 0.0285 | | 0.0008 | 0.7153 | | 0.2537 | G |
| 10292 | 0.0001 | 0.0429 | | 0.0007 | 0.952 | | 0.004 | G |
| 10295 | 0.0002 | 0.203 | | 0.4495 | 0.0375 | | 0.3096 | C |
| 10298 | 0.0002 | 0.2168 | | 0.0088 | 0.5635 | | 0.2106 | G |
| 10306 | 0.0001 | 0.0002 | | 0.9959 | 0.0005 | | 0.0031 | C |
| 10408 | 0.0001 | 0.001 | | 0.0003 | 0.9978 | | 0.0003 | G |
| 10413 | 0.0001 | 0.9981 | | 0.0001 | 0.0013 | | 0.0002 | A |
| 10422 | 0.0001 | 0.991 | | 0.0036 | 0.0049 | | 0.0003 | A |
| 10458 | 0.0005 | 0.9819 | | 0.0001 | 0.017 | | 0.0002 | A |
| 10463 | 0.0002 | 0.0005 | | 0.0002 | 0.9988 | | 0.0001 | G |
| 10467 | 0.0002 | 0.0001 | | 0.9332 | 0.0023 | | 0.064 | C |
| 10475 | 0.0002 | 0.1294 | | 0.0207 | 0.8187 | | 0.0308 | G |
| 10486 | 0.0004 | 0.0447 | | 0.0178 | 0.0071 | | 0.9299 | T |
| 10496 | 0.0001 | 0.0036 | | 0.0001 | 0.996 | | 0.0002 | G |
| 10500 | 0.0002 | 0.0006 | | 0.0001 | 0.9987 | | 0.0002 | G |
| 10506 | 0.0018 | 0.0005 | | 0.0001 | 0.9972 | | 0.0002 | G |
| 10562 | 0.0001 | 0.0009 | | 0.0004 | 0.7801 | | 0.218 | G |
| 10620 | 0.0002 | 0.9981 | | 0.0001 | 0.0014 | | 0.0001 | A |
| 10628 | 0.0001 | 0.0006 | | 0.0005 | 0.799 | | 0.1997 | G |
| 10634 | 0.0001 | 0.0006 | | 0.9973 | 0.0001 | | 0.0017 | C |
| 10642 | 0.0012 | 0.6972 | | 0.0008 | 0.3004 | | 0.0001 | A |
| 10701 | 0.0001 | 0.9963 | | 0.0005 | 0.0015 | | 0.0003 | A |
| 10719 | 0.0002 | 0.9969 | | 0.0009 | 0.0014 | | 0.0003 | A |
| 10732 | 0.0001 | 0.0082 | | 0.9698 | 0.0062 | | 0.0155 | C |
| 10742 | 0.0002 | 0.9464 | | 0.0062 | 0.0466 | | 0.0004 | A |
| 10748 | 0.0003 | 0.0025 | | 0.0001 | 0.9967 | | 0.0001 | G |
| 10753 | 0.0001 | 0.0022 | | 0.0001 | 0.997 | | 0.0001 | G |
| 10763 | 0.0002 | 0.9363 | | 0.0616 | 0.0014 | | 0.0004 | A |
| 10833 | 0.0002 | 0.0004 | | 0.0009 | 0.0007 | | 0.9976 | T |
| 10839 | 0.0005 | 0.0003 | | 0.0005 | 0.0002 | | 0.9984 | T |
| 10849 | 0.0002 | 0.9982 | | 0.0002 | 0.0011 | | 0.0002 | A |
| 10858 | 0.0002 | 0.0009 | | 0.0003 | 0.9981 | | 0.0003 | G |
| 10865 | 0.0001 | 0.9982 | | 0.0001 | 0.0014 | | 0.0002 | A |
| 10873 | 0.0001 | 0.0012 | | 0.0008 | 0.002 | | 0.9959 | T |
| 10883 | 0.0002 | 0.998 | | 0.0006 | 0.0009 | | 0.0003 | A |
| 10888 | 0.0011 | 0.0001 | | 0.9981 | 0.0001 | | 0.0005 | C |
| 10923 | 0.0002 | 0.0002 | | 0.9978 | 0.0002 | | 0.0011 | C |
| 10927 | 0.0001 | 0.0002 | | 0.9976 | 0.0004 | | 0.0013 | C |
| 10931 | 0.0002 | 0.0006 | | 0.0465 | 0.0063 | | 0.9463 | T |
| 10936 | 0.0001 | 0.0156 | | 0.0062 | 0.97 | | 0.0078 | G |
| 10946 | 0.0005 | 0.0005 | | 0.0002 | 0.9982 | | 0.0003 | G |
| 10952 | 0.0001 | 0.0001 | | 0.001 | 0.0001 | | 0.9985 | T |
| 10955 | 0.0001 | 0.9984 | | 0.0001 | 0.0011 | | 0.0002 | A |
| 11005 | 0.0001 | 0.0013 | | 0.0002 | 0.9976 | | 0.0007 | G |
| 11010 | 0.0001 | 0.0001 | | 0.0014 | 0.0001 | | 0.9982 | T |
| 11034 | 0.001 | 0.0002 | | 0.9973 | 0.0004 | | 0.0008 | C |
| 11038 | 0.0001 | 0.0001 | | 0.9952 | 0 | | 0.0042 | C |
| 11044 | 0.0002 | 0.929 | | 0.0074 | 0.0184 | | 0.0448 | A |
| 11048 | 0.0002 | 0.0306 | | 0.8181 | 0.0206 | | 0.1304 | C |
| 11054 | 0.0002 | 0.0638 | | 0.003 | 0.9326 | | 0.0002 | G |
| 11058 | 0.0001 | 0.0001 | | 0.999 | 0.0001 | | 0.0006 | C |
| 11066 | 0.0003 | 0.0354 | | 0.796 | 0.0264 | | 0.1415 | C |
| 11074 | 0.0003 | 0.003 | | 0.1019 | 0.8923 | | 0.0024 | G |
| 11084 | 0.0002 | 0.0002 | | 0.0012 | 0.0002 | | 0.9981 | T |
| 11090 | 0.0011 | 0.9973 | | 0.0005 | 0.0006 | | 0.0003 | A |
| 11107 | 0.0001 | 0.9975 | | 0.0002 | 0.0011 | | 0.0002 | A |
| 11110 | 0.0001 | 0.9973 | | 0.0006 | 0.0015 | | 0.0002 | A |
| 11117 | 0.0002 | 0.0002 | | 0.9987 | 0.0002 | | 0.0007 | C |
| 11127 | 0.0002 | 0.0021 | | 0.001 | 0.9895 | | 0.007 | G |
| 11139 | 0.0002 | 0.7973 | | 0.0069 | 0.1525 | | 0.043 | A |
| 11146 | 0.0003 | 0.0009 | | 0.0015 | 0.0007 | | 0.9966 | T |
| 11153 | 0.0003 | 0.0009 | | 0.0025 | 0.9961 | | 0.0002 | G |
| 11157 | 0.0004 | 0.5026 | | 0.0325 | 0.2708 | | 0.1935 | A |
| 11162 | 0.0005 | 0.1951 | | 0.1884 | 0.5313 | | 0.0845 | G |
| 11169 | 0.0005 | 0.1374 | | 0.1636 | 0.0585 | | 0.6399 | T |
| 11174 | 0.0003 | 0.5316 | | 0.0003 | 0.4672 | | 0.0004 | A |
| 11179 | 0.0003 | 0.0022 | | 0.9394 | 0.001 | | 0.0569 | C |
| 11187 | 0.0003 | 0.0009 | | 0.0429 | 0.0007 | | 0.9553 | T |
| 11199 | 0.0003 | 0.8206 | | 0.0642 | 0.0569 | | 0.058 | A |
| 11204 | 0.0004 | 0.2128 | | 0.0006 | 0.785 | | 0.0009 | G |
| 11210 | 0.0002 | 0.0556 | | 0.1526 | 0.6824 | | 0.1091 | G |
| 11215 | 0.0006 | 0.0389 | | 0.1187 | 0.0136 | | 0.8281 | T |
| 11220 | 0.0003 | 0.0127 | | 0.0036 | 0.9661 | | 0.0172 | G |
| 11225 | 0.0002 | 0.005 | | 0.0154 | 0.044 | | 0.9353 | T |
| 11231 | 0.0003 | 0.0605 | | 0.1265 | 0.2172 | | 0.5953 | T |
| 11234 | 0.0002 | 0.27 | | 0.0334 | 0.6091 | | 0.0872 | G |
| 11237 | 0.0001 | 0.0052 | | 0.02 | 0.968 | | 0.0066 | G |
| 11240 | 0.0009 | 0.1586 | | 0.081 | 0.6601 | | 0.0993 | G |
| 11241 | 0.0008 | 0.1207 | | 0.1485 | 0.5498 | | 0.18 | G |
| 11242 | 0.0001 | 0.1522 | | 0.2763 | 0.4331 | | 0.1381 | G |
| 11243 | 0.0009 | 0.0772 | | 0.3663 | 0.3194 | | 0.2361 | C |
| 11244 | 0.0058 | 0.2314 | | 0.2197 | 0.0569 | | 0.486 | T |
| 11245 | 0.0074 | 0.1523 | | 0.0607 | 0.0296 | | 0.7498 | T |
| 11246 | 0.0147 | 0.1887 | | 0.1178 | 0.0629 | | 0.6156 | T |
| 11247 | 0.0249 | 0.1725 | | 0.4659 | 0.2283 | | 0.1083 | C |
| 11248 | 0.1148 | 0.2373 | | 0.4643 | 0.1106 | | 0.0729 | C |
| 11249 | 0.1377 | 0.1993 | | 0.174 | 0.3966 | | 0.0922 | G |
| 11250 | 0.2486 | 0.0435 | | 0.46 | 0.1895 | | 0.0583 | C |
| 12118 | 0.0008 | 0.1299 | | 0.4647 | 0.2306 | | 0.1738 | C |
| 12119 | 0.0008 | 0.0473 | | 0.3872 | 0.1351 | | 0.4293 | T |
| 12122 | 0.0001 | 0.0021 | | 0.0434 | 0.0311 | | 0.9231 | T |
| 12125 | 0.0004 | 0.0286 | | 0.5103 | 0.0939 | | 0.3667 | C |
| 12128 | 0.0004 | 0.5139 | | 0.1969 | 0.2055 | | 0.0832 | A |
| 12133 | 0.0004 | 0.0007 | | 0.043 | 0.948 | | 0.0078 | G |
| 12137 | 0.0002 | 0.0159 | | 0.0859 | 0.0144 | | 0.8835 | T |
| 12142 | 0.0002 | 0.0941 | | 0.013 | 0.8509 | | 0.0417 | G |
| 12147 | 0.0003 | 0.1078 | | 0.6733 | 0.1538 | | 0.0647 | C |
| 12151 | 0.0002 | 0.0008 | | 0.7749 | 0.0009 | | 0.2231 | C |
| 12161 | 0.0002 | 0.024 | | 0.0536 | 0.8907 | | 0.0313 | G |
| 12170 | 0.0002 | 0.1774 | | 0.4855 | 0.0799 | | 0.2568 | C |
| 12180 | 0.0002 | 0.9596 | | 0.0384 | 0.0014 | | 0.0003 | A |
| 12183 | 0.0006 | 0.0016 | | 0.0121 | 0.985 | | 0.0003 | G |
| 12189 | 0.0006 | 0.002 | | 0.8867 | 0.0063 | | 0.1039 | C |
| 12205 | 0.0002 | 0.0674 | | 0.0149 | 0.0943 | | 0.8231 | T |
| 12216 | 0.0004 | 0.9975 | | 0.0008 | 0.0007 | | 0.0005 | A |
| 12221 | 0.0001 | 0.9982 | | 0.0003 | 0.0013 | | 0.0002 | A |
| 12225 | 0.0002 | 0.0951 | | 0.9019 | 0.0004 | | 0.0023 | C |
| 12237 | 0.0002 | 0.0456 | | 0.0058 | 0.9465 | | 0.0018 | G |
| 12242 | 0.0001 | 0.0002 | | 0.9422 | 0.0113 | | 0.0462 | C |
| 12247 | 0.0003 | 0.6516 | | 0.0014 | 0.2803 | | 0.0663 | A |
| 12257 | 0.0003 | 0.1863 | | 0.0086 | 0.0269 | | 0.7779 | T |
| 12261 | 0.0001 | 0.0005 | | 0.0006 | 0.0708 | | 0.9278 | T |
| 12265 | 0.0003 | 0.994 | | 0.0001 | 0.0046 | | 0.0009 | A |
| 12272 | 0.0001 | 0.9982 | | 0.0001 | 0.0014 | | 0.0002 | A |
| 12282 | 0.0003 | 0.055 | | 0.0005 | 0.9438 | | 0.0003 | G |
| 12291 | 0.0002 | 0.0002 | | 0.4675 | 0.0001 | | 0.532 | T |
| 12298 | 0.0003 | 0.6403 | | 0.0586 | 0.1635 | | 0.1372 | A |
| 12303 | 0.0003 | 0.0844 | | 0.531 | 0.1885 | | 0.1956 | C |
| 12307 | 0.0003 | 0.1932 | | 0.271 | 0.0323 | | 0.5032 | T |
| 12311 | 0.0001 | 0.0001 | | 0.9969 | 0.0019 | | 0.0009 | C |
| 12315 | 0.0001 | 0.0001 | | 0.9987 | 0.0001 | | 0.0007 | C |
| 12350 | 0.0002 | 0.1688 | | 0.0011 | 0.8293 | | 0.0004 | G |
| 12353 | 0.0001 | 0.0001 | | 0.9991 | 0.0002 | | 0.0005 | C |
| 12358 | 0.0006 | 0.0004 | | 0.9968 | 0.0006 | | 0.0013 | C |
| 12385 | 0.0001 | 0.0003 | | 0.0016 | 0.0007 | | 0.9973 | T |
| 12392 | 0.0001 | 0.0004 | | 0.0001 | 0.9992 | | 0.0002 | G |
| 12398 | 0.0001 | 0.0081 | | 0.0001 | 0.9896 | | 0.0022 | G |
| 12402 | 0.0001 | 0.0006 | | 0.0001 | 0.999 | | 0.0001 | G |
| 12411 | 0.0012 | 0.0174 | | 0.0001 | 0.9747 | | 0.0066 | G |
| 12419 | 0.0001 | 0.9977 | | 0 | 0.0019 | | 0.0002 | A |
| 12427 | 0.0001 | 0.0008 | | 0.0078 | 0.9847 | | 0.0066 | G |
| 12440 | 0.0001 | 0.0002 | | 0.0008 | 0.0003 | | 0.9986 | T |
| 12445 | 0.0001 | 0.9979 | | 0.0002 | 0.0014 | | 0.0002 | A |
| 12453 | 0.0001 | 0.0001 | | 0.9844 | 0.0003 | | 0.0151 | C |
| 12457 | 0.0001 | 0.002 | | 0.0002 | 0.9974 | | 0.0002 | G |
| 12461 | 0.0002 | 0.2847 | | 0.0983 | 0.5389 | | 0.0777 | G |
| 12467 | 0.0002 | 0.0271 | | 0.7035 | 0.006 | | 0.263 | C |
| 12470 | 0.0001 | 0.0002 | | 0.9468 | 0.0515 | | 0.0013 | C |
| 12477 | 0.0001 | 0.0013 | | 0.0004 | 0.998 | | 0.0001 | G |
| 12490 | 0.0001 | 0.0001 | | 0.9994 | 0.0001 | | 0.0004 | C |
| 12496 | 0.0001 | 0.9981 | | 0.0003 | 0.0012 | | 0.0002 | A |
| 12512 | 0.0003 | 0.9974 | | 0.0002 | 0.0017 | | 0.0003 | A |
| 12562 | 0.0001 | 0.0007 | | 0.0512 | 0.9479 | | 0.0001 | G |
| 12565 | 0.0003 | 0.2339 | | 0.0059 | 0.7326 | | 0.0273 | G |
| 12573 | 0.0005 | 0.0776 | | 0.2839 | 0.0982 | | 0.5397 | T |
| 12577 | 0.0002 | 0.0004 | | 0.0013 | 0.0004 | | 0.9977 | T |
| 12581 | 0.0006 | 0.433 | | 0.0001 | 0.564 | | 0.0021 | G |
| 12586 | 0.0003 | 0.9978 | | 0.0001 | 0.0016 | | 0.0002 | A |
| 12588 | 0.0001 | 0.9981 | | 0.0001 | 0.0014 | | 0.0002 | A |
| 12592 | 0.0001 | 0.9979 | | 0.0003 | 0.0016 | | 0.0001 | A |
| 12652 | 0.0002 | 0.0002 | | 0.9987 | 0.0001 | | 0.0007 | C |
| 12655 | 0.0001 | 0.0022 | | 0.0017 | 0.0001 | | 0.9959 | T |
| 12660 | 0.0002 | 0.0001 | | 0.9947 | 0 | | 0.0049 | C |
| 12673 | 0 | 0.9985 | | 0.0002 | 0.001 | | 0.0001 | A |
| 12682 | 0.0001 | 0.9984 | | 0.0001 | 0.0012 | | 0.0002 | A |
| 12690 | 0.0005 | 0.9973 | | 0.0001 | 0.0018 | | 0.0002 | A |
| 12701 | 0.0002 | 0.0079 | | 0.0006 | 0.8781 | | 0.113 | G |
| 12709 | 0.0001 | 0.0224 | | 0 | 0.9772 | | 0.0002 | G |
| 12718 | 0.0001 | 0.9984 | | 0 | 0.0013 | | 0.0001 | A |
| 12737 | 0.0002 | 0.9984 | | 0 | 0.0012 | | 0.0002 | A |
| 12753 | 0.0003 | 0.0005 | | 0.0008 | 0.0001 | | 0.9983 | T |
| 12757 | 0.0001 | 0.0067 | | 0.0012 | 0.0005 | | 0.9915 | T |
| 12761 | 0.0001 | 0.0003 | | 0 | 0.9994 | | 0.0001 | G |
| 12764 | 0.0001 | 0.9942 | | 0.0002 | 0.0052 | | 0.0002 | A |
| 12772 | 0.0001 | 0.0001 | | 0.9991 | 0.0002 | | 0.0004 | C |
| 12776 | 0.0001 | 0.0008 | | 0.0001 | 0.9987 | | 0.0001 | G |
| 12816 | 0.0013 | 0.0004 | | 0.0001 | 0.9981 | | 0.0001 | G |
| 12823 | 0.0001 | 0.0005 | | 0.0001 | 0.9992 | | 0.0001 | G |
| 12828 | 0.0001 | 0.004 | | 0.0009 | 0.9939 | | 0.0009 | G |
| 12838 | 0.0002 | 0.3469 | | 0.0633 | 0.5891 | | 0.0004 | G |
| 12847 | 0.0011 | 0.0003 | | 0.988 | 0.0007 | | 0.0096 | C |
| 12865 | 0.0001 | 0.0001 | | 0.9926 | 0.0003 | | 0.0068 | C |
| 12871 | 0.0001 | 0.0002 | | 0.9895 | 0.0001 | | 0.0097 | C |
| 12877 | 0.0001 | 0.0064 | | 0.0004 | 0.9928 | | 0.0001 | G |
| 12886 | 0.0001 | 0.0004 | | 0.999 | 0 | | 0.0005 | C |
| 12895 | 0.0001 | 0.9975 | | 0.0006 | 0.0015 | | 0.0002 | A |
| 12898 | 0.0001 | 0 | | 0.9995 | 0 | | 0.0004 | C |
| 12904 | 0.0002 | 0.9981 | | 0.0003 | 0.0011 | | 0.0001 | A |
| 12937 | 0.0001 | 0.9977 | | 0.0002 | 0.0017 | | 0.0002 | A |
| 12945 | 0.0001 | 0.0005 | | 0.0015 | 0.9975 | | 0.0002 | G |
| 12949 | 0.0001 | 0.0002 | | 0.9858 | 0.0001 | | 0.0137 | C |
| 12959 | 0.0001 | 0.0795 | | 0.0002 | 0.9201 | | 0.0001 | G |
| 12968 | 0.0003 | 0.0004 | | 0.0001 | 0.999 | | 0.0001 | G |
| 12972 | 0.0001 | 0.0556 | | 0.2234 | 0.0004 | | 0.7205 | T |
| 12980 | 0.0001 | 0.0004 | | 0.0001 | 0.9992 | | 0.0001 | G |
| 12990 | 0.0001 | 0.0005 | | 0.0001 | 0.9992 | | 0.0001 | G |
| 12995 | 0.0001 | 0.9973 | | 0.0001 | 0.0019 | | 0.0006 | A |
| 13000 | 0.0001 | 0.0552 | | 0.0002 | 0.8503 | | 0.0939 | G |
| 13007 | 0.0001 | 0.0001 | | 0.8483 | 0.0692 | | 0.0823 | C |
| 13015 | 0.0001 | 0.99 | | 0.0001 | 0.0096 | | 0.0001 | A |
| 13036 | 0.0001 | 0.0002 | | 0.0032 | 0.0001 | | 0.9963 | T |
| 13040 | 0.0001 | 0.0005 | | 0.001 | 0.9983 | | 0.0001 | G |
| 13051 | 0.0001 | 0.0003 | | 0.0949 | 0.0005 | | 0.9042 | T |
| 13055 | 0.0002 | 0.0004 | | 0.0001 | 0.9885 | | 0.0106 | G |
| 13059 | 0.0001 | 0.0004 | | 0.0027 | 0.9966 | | 0.0002 | G |
| 13062 | 0.0001 | 0.2108 | | 0.0296 | 0.0004 | | 0.759 | T |
| 13071 | 0.0004 | 0.0002 | | 0.0012 | 0.0002 | | 0.9978 | T |
| 13099 | 0.0001 | 0.0003 | | 0.0117 | 0.0001 | | 0.9878 | T |
| 13110 | 0.0001 | 0.999 | | 0 | 0.0005 | | 0.0002 | A |
| 13114 | 0.0003 | 0.9984 | | 0.0001 | 0.0007 | | 0.0004 | A |
| 13118 | 0.0001 | 0.0001 | | 0.0013 | 0 | | 0.9984 | T |
| 13124 | 0.0001 | 0.0005 | | 0.0031 | 0.0001 | | 0.9962 | T |
| 13132 | 0.0001 | 0.0008 | | 0.9929 | 0.001 | | 0.0048 | C |
| 13142 | 0.0001 | 0.0007 | | 0.0002 | 0.9987 | | 0.0002 | G |
| 13153 | 0.0001 | 0.9972 | | 0.0001 | 0.001 | | 0.0016 | A |
| 13158 | 0.0002 | 0.4425 | | 0.0781 | 0.0102 | | 0.4689 | T |
| 13163 | 0.0001 | 0.0033 | | 0.0002 | 0.9959 | | 0.0001 | G |
| 13167 | 0.0001 | 0.1035 | | 0.8918 | 0.0028 | | 0.0011 | C |
| 13171 | 0.0003 | 0.874 | | 0.0004 | 0.003 | | 0.1222 | A |
| 13179 | 0.0001 | 0.9972 | | 0.0004 | 0.002 | | 0.0001 | A |
| 13186 | 0.0001 | 0.0085 | | 0.9908 | 0.0001 | | 0.0005 | C |
| 13189 | 0.0001 | 0.0068 | | 0.0059 | 0.9869 | | 0.0002 | G |
| 13219 | 0.0001 | 0.0003 | | 0.9986 | 0.0003 | | 0.0006 | C |
| 13222 | 0.0002 | 0.0005 | | 0.0004 | 0.9988 | | 0 | G |
| 13230 | 0.0001 | 0.8869 | | 0.0544 | 0.0026 | | 0.0561 | A |
| 13238 | 0.0002 | 0.9029 | | 0.0001 | 0.0959 | | 0.0009 | A |
| 13244 | 0.0002 | 0.0702 | | 0.0005 | 0.9286 | | 0.0003 | G |
| 13250 | 0.0003 | 0.9976 | | 0.0002 | 0.0017 | | 0.0001 | A |
| 13257 | 0.0001 | 0.9968 | | 0.0007 | 0.0021 | | 0.0002 | A |
| 13269 | 0.0001 | 0.0001 | | 0.9991 | 0 | | 0.0006 | C |
| 13280 | 0.0003 | 0.0002 | | 0.9988 | 0.0001 | | 0.0006 | C |
| 13286 | 0.0001 | 0.0002 | | 0.0014 | 0.0001 | | 0.9982 | T |
| 13292 | 0.0003 | 0.0001 | | 0.0028 | 0 | | 0.9967 | T |
| 13300 | 0.0001 | 0.9983 | | 0.0004 | 0.0009 | | 0.0003 | A |
| 13305 | 0.0002 | 0.0001 | | 0.999 | 0.0001 | | 0.0006 | C |
| 13310 | 0.0001 | 0.0001 | | 0.9991 | 0.0001 | | 0.0005 | C |
| 13314 | 0.0001 | 0.5358 | | 0.0839 | 0.0036 | | 0.3767 | A |
| 13318 | 0.0002 | 0.403 | | 0.0042 | 0.5883 | | 0.0043 | G |
| 13322 | 0.0001 | 0.1566 | | 0.1641 | 0.6763 | | 0.0029 | G |
| 13327 | 0.0002 | 0.0446 | | 0.1555 | 0.3565 | | 0.4431 | T |
| 13332 | 0.0002 | 0.005 | | 0.8552 | 0.0001 | | 0.1394 | C |
| 13338 | 0.0005 | 0.0003 | | 0.0014 | 0.0002 | | 0.9976 | T |
| 13345 | 0.0005 | 0.0096 | | 0.0184 | 0.0013 | | 0.9702 | T |
| 13359 | 0.0005 | 0.0476 | | 0.0001 | 0.9515 | | 0.0003 | G |
| 13371 | 0.0006 | 0.9942 | | 0.0003 | 0.0046 | | 0.0003 | A |
| 13380 | 0.0006 | 0.0998 | | 0.8962 | 0.0009 | | 0.0025 | C |
| 13384 | 0.0006 | 0.9118 | | 0.0264 | 0.0027 | | 0.0585 | A |
| 13389 | 0.0006 | 0.0024 | | 0.0029 | 0.0526 | | 0.9415 | T |
| 13400 | 0.0008 | 0.1626 | | 0.4863 | 0.3157 | | 0.0346 | C |
| 13406 | 0.0002 | 0.2008 | | 0.5471 | 0.1075 | | 0.1443 | C |
| 13416 | 0.0002 | 0.2342 | | 0.3151 | 0.0672 | | 0.3831 | T |
| 13422 | 0.0001 | 0.1485 | | 0.3224 | 0.2193 | | 0.3094 | C |
| 13426 | 0.0001 | 0.1731 | | 0.1815 | 0.3226 | | 0.3223 | G |
| 13427 | 0.0001 | 0.0311 | | 0.0091 | 0.7767 | | 0.183 | G |
| 13444 | 0.0005 | 0.8072 | | 0.0813 | 0.0824 | | 0.0284 | A |
| 13445 | 0.0002 | 0.4754 | | 0.3743 | 0.0473 | | 0.1026 | A |
| 13446 | 0.0001 | 0.2462 | | 0.3976 | 0.1928 | | 0.163 | C |
| 13447 | 0.0003 | 0.2004 | | 0.2205 | 0.3537 | | 0.2249 | G |
| 13450 | 0.0003 | 0.1114 | | 0.4576 | 0.1793 | | 0.2511 | C |
| 13453 | 0.0003 | 0.0943 | | 0.2334 | 0.105 | | 0.5666 | T |
| 13457 | 0.0006 | 0.063 | | 0.5081 | 0.222 | | 0.206 | C |
| 13462 | 0.0006 | 0.0556 | | 0.4035 | 0.064 | | 0.476 | T |
| 13465 | 0.0006 | 0.7895 | | 0.0215 | 0.1097 | | 0.0786 | A |
| 13474 | 0.0008 | 0.0006 | | 0.0038 | 0.9411 | | 0.0536 | G |
| 13481 | 0.0007 | 0.9881 | | 0.0012 | 0.0016 | | 0.0084 | A |
| 13486 | 0.0006 | 0.2091 | | 0.0001 | 0.79 | | 0.0002 | G |
| 13490 | 0.0042 | 0.9413 | | 0.02 | 0.0043 | | 0.0302 | A |
| 13496 | 0.0004 | 0.0062 | | 0.1189 | 0.0965 | | 0.778 | T |
| 13499 | 0.0003 | 0.4603 | | 0.0557 | 0.4182 | | 0.0652 | A |
| 13503 | 0.0003 | 0.0974 | | 0.1318 | 0.6173 | | 0.1528 | G |
| 13508 | 0.0003 | 0.4645 | | 0.0527 | 0.3384 | | 0.1437 | A |
| 13511 | 0.0003 | 0.1319 | | 0.0942 | 0.491 | | 0.2823 | G |
| 13513 | 0.0002 | 0.0967 | | 0.2837 | 0.2334 | | 0.3855 | T |
| 13514 | 0.0002 | 0.1628 | | 0.1767 | 0.1895 | | 0.4704 | T |
| 13515 | 0 | 0.0166 | | 0.0719 | 0.3892 | | 0.522 | T |
| 13516 | 0.0001 | 0.0238 | | 0.299 | 0.0302 | | 0.6468 | T |
| 13517 | 0.0001 | 0.0237 | | 0.6177 | 0.2407 | | 0.1176 | C |
| 13518 | 0.0001 | 0.0201 | | 0.7709 | 0.0552 | | 0.1535 | C |
| 13519 | 0.0001 | 0.0353 | | 0.5538 | 0.0276 | | 0.383 | C |
| 13520 | 0.0003 | 0.0345 | | 0.3075 | 0.0565 | | 0.601 | T |
| 13521 | 0.0006 | 0.0585 | | 0.2122 | 0.1639 | | 0.5648 | T |
| 13522 | 0.0011 | 0.086 | | 0.2667 | 0.2965 | | 0.3494 | T |
| 13523 | 0.0115 | 0.1264 | | 0.2047 | 0.5141 | | 0.1432 | G |
| 13524 | 0.0587 | 0.1142 | | 0.0311 | 0.7529 | | 0.0429 | G |
| 13525 | 0.1098 | 0.1459 | | 0.0192 | 0.6901 | | 0.035 | G |
| 13526 | 0.2266 | 0.2198 | | 0.1484 | 0.3736 | | 0.0315 | G |
| 14558 | 0.0001 | 0.1714 | | 0.6754 | 0.0247 | | 0.1284 | C |
| 14559 | 0.0002 | 0.3009 | | 0.2497 | 0.2017 | | 0.2472 | A |
| 14562 | 0.0003 | 0.2603 | | 0.208 | 0.3703 | | 0.1609 | G |
| 14565 | 0.0003 | 0.3153 | | 0.0632 | 0.4017 | | 0.2193 | G |
| 14570 | 0.0004 | 0.1565 | | 0.0635 | 0.5457 | | 0.2336 | G |
| 14573 | 0.0723 | 0.4121 | | 0.0041 | 0.1036 | | 0.4078 | A |
| 14580 | 0.0012 | 0.0343 | | 0.2391 | 0.487 | | 0.2384 | G |
| 14588 | 0.0006 | 0.8949 | | 0.0498 | 0.0503 | | 0.0044 | A |
| 14592 | 0.001 | 0.0574 | | 0.9017 | 0.0271 | | 0.0128 | C |
| 14600 | 0.0001 | 0.9974 | | 0.0002 | 0.0018 | | 0.0004 | A |
| 14604 | 0.0002 | 0.0006 | | 0.0002 | 0.9989 | | 0.0001 | G |
| 14609 | 0.0001 | 0.0023 | | 0.0003 | 0.9971 | | 0.0002 | G |
| 14625 | 0.0001 | 0.0002 | | 0.0018 | 0.0002 | | 0.9976 | T |
| 14632 | 0.0001 | 0.0009 | | 0.0001 | 0.9988 | | 0.0001 | G |
| 14636 | 0.0002 | 0.0025 | | 0.3838 | 0.6069 | | 0.0065 | G |
| 14640 | 0.0001 | 0.0002 | | 0.0018 | 0.0002 | | 0.9975 | T |
| 14644 | 0.0001 | 0.0005 | | 0.0001 | 0.9992 | | 0.0001 | G |
| 14653 | 0.0001 | 0.0001 | | 0.9993 | 0 | | 0.0005 | C |
| 14664 | 0.0001 | 0.998 | | 0.0001 | 0.0016 | | 0.0002 | A |
| 14669 | 0.0001 | 0.0002 | | 0.0138 | 0.0001 | | 0.9858 | T |
| 14672 | 0.0002 | 0.0004 | | 0 | 0.9992 | | 0.0001 | G |
| 14677 | 0.0001 | 0.0005 | | 0.0063 | 0.993 | | 0.0001 | G |
| 14680 | 0.0002 | 0.0001 | | 0.441 | 0.0001 | | 0.5586 | T |
| 14695 | 0.0002 | 0.0004 | | 0.0234 | 0.0003 | | 0.9756 | T |
| 14700 | 0.0001 | 0.0004 | | 0.0001 | 0.9994 | | 0.0001 | G |
| 14703 | 0.0001 | 0.0002 | | 0.0015 | 0 | | 0.9981 | T |
| 14707 | 0.0001 | 0.0002 | | 0.9982 | 0.0002 | | 0.0013 | C |
| 14710 | 0.0001 | 0.0005 | | 0.0002 | 0.9989 | | 0.0002 | G |
| 14716 | 0.0001 | 0.0017 | | 0.0014 | 0.0001 | | 0.9966 | T |
| 14724 | 0.0001 | 0.0001 | | 0.9994 | 0 | | 0.0004 | C |
| 14734 | 0.0001 | 0.9981 | | 0.0001 | 0.0014 | | 0.0002 | A |
| 14746 | 0.0001 | 0.0005 | | 0.0002 | 0.9991 | | 0.0001 | G |
| 14750 | 0.0001 | 0.0001 | | 0.9977 | 0 | | 0.002 | C |
| 14756 | 0.0001 | 0.0003 | | 0.0015 | 0 | | 0.9981 | T |
| 14761 | 0.0001 | 0.0002 | | 0.9984 | 0.0001 | | 0.0011 | C |
| 14766 | 0.0001 | 0.0005 | | 0.0001 | 0.9992 | | 0.0001 | G |
| 14774 | 0.0001 | 0.0002 | | 0.0017 | 0.0001 | | 0.9978 | T |
| 14781 | 0.0001 | 0.0005 | | 0.0001 | 0.9991 | | 0.0002 | G |
| 14785 | 0.0001 | 0.0003 | | 0.1047 | 0.0002 | | 0.8947 | T |
| 14790 | 0.0001 | 0.0001 | | 0.9494 | 0.0002 | | 0.0503 | C |
| 14808 | 0.0001 | 0.0007 | | 0.0001 | 0.9982 | | 0.0008 | G |
| 14815 | 0.0001 | 0.0002 | | 0.0014 | 0.0002 | | 0.9981 | T |
| 14820 | 0.0001 | 0.0021 | | 0 | 0.9976 | | 0.0001 | G |
| 14832 | 0.0001 | 0.9976 | | 0.0001 | 0.002 | | 0.0002 | A |
| 14853 | 0.0001 | 0.0459 | | 0.0001 | 0.9537 | | 0 | G |
| 14856 | 0.0001 | 0.894 | | 0.0002 | 0.1054 | | 0.0003 | A |
| 14860 | 0.0001 | 0.0003 | | 0.002 | 0.0001 | | 0.9975 | T |
| 14868 | 0.0001 | 0.0004 | | 0.0001 | 0.9993 | | 0.0001 | G |
| 14876 | 0.0002 | 0.0003 | | 0.0014 | 0.0001 | | 0.998 | T |
| 14881 | 0.0001 | 0.0013 | | 0.0675 | 0.0003 | | 0.9308 | T |
| 14888 | 0.0002 | 0.0257 | | 0.017 | 0.9506 | | 0.0065 | G |
| 14891 | 0.0001 | 0.0016 | | 0.003 | 0.995 | | 0.0003 | G |
| 14895 | 0.0001 | 0.0005 | | 0.0497 | 0.9493 | | 0.0003 | G |
| 14901 | 0.0003 | 0.0002 | | 0.0014 | 0.0002 | | 0.998 | T |
| 14909 | 0.0001 | 0.0001 | | 0.0055 | 0 | | 0.9941 | T |
| 14917 | 0.0002 | 0.9975 | | 0.0011 | 0.0008 | | 0.0002 | A |
| 14922 | 0.0001 | 0.998 | | 0.0001 | 0.0016 | | 0.0002 | A |
| 14927 | 0.0001 | 0.0003 | | 0.0001 | 0.9992 | | 0.0001 | G |
| 14931 | 0.0001 | 0.0001 | | 0.0015 | 0.0001 | | 0.9982 | T |
| 14952 | 0.0002 | 0.0002 | | 0.9489 | 0.0497 | | 0.0009 | C |
| 14959 | 0.0001 | 0.0003 | | 0.9942 | 0.003 | | 0.0024 | C |
| 14968 | 0.0001 | 0.0056 | | 0.9492 | 0.0168 | | 0.0283 | C |
| 14972 | 0.0001 | 0.072 | | 0.0025 | 0.9186 | | 0.0067 | G |
| 14979 | 0.0001 | 0.0019 | | 0.8766 | 0.0004 | | 0.1209 | C |
| 14983 | 0.0002 | 0.9983 | | 0.0001 | 0.0012 | | 0.0001 | A |
| 14990 | 0.0001 | 0.9982 | | 0.0002 | 0.0014 | | 0.0001 | A |
| 14993 | 0.0002 | 0.0001 | | 0.9991 | 0.0001 | | 0.0005 | C |
| 15004 | 0.0001 | 0.0006 | | 0.0001 | 0.9991 | | 0 | G |
| 15011 | 0.0001 | 0.9983 | | 0 | 0.0014 | | 0.0002 | A |
| 15015 | 0.0001 | 0.0016 | | 0 | 0.9982 | | 0 | G |
| 15027 | 0.0001 | 0.0001 | | 0.999 | 0.0001 | | 0.0007 | C |
| 15045 | 0.0001 | 0.0007 | | 0.0001 | 0.9991 | | 0 | G |
| 15056 | 0.0001 | 0.0054 | | 0.9921 | 0.0001 | | 0.0023 | C |
| 15066 | 0.0001 | 0.9981 | | 0.0002 | 0.0014 | | 0.0002 | A |
| 15076 | 0.0002 | 0.9976 | | 0.0003 | 0.0016 | | 0.0002 | A |
| 15087 | 0.0003 | 0.0001 | | 0.9991 | 0.0001 | | 0.0004 | C |
| 15092 | 0.0001 | 0.0001 | | 0.999 | 0.0001 | | 0.0007 | C |
| 15097 | 0.0001 | 0.0003 | | 0.9984 | 0.0001 | | 0.0011 | C |
| 15101 | 0.0001 | 0.0163 | | 0.2388 | 0.0008 | | 0.7439 | T |
| 15105 | 0.0001 | 0.0004 | | 0.1206 | 0.0003 | | 0.8785 | T |
| 15116 | 0.0001 | 0.4613 | | 0.009 | 0.4953 | | 0.0342 | G |
| 15119 | 0.0001 | 0.1644 | | 0.1125 | 0.0012 | | 0.7217 | T |
| 15123 | 0.0001 | 0.0182 | | 0.5297 | 0.1732 | | 0.2786 | C |
| 15130 | 0.0001 | 0.1255 | | 0.4989 | 0.1744 | | 0.201 | C |
| 15133 | 0.0001 | 0.0763 | | 0.1178 | 0.0103 | | 0.7954 | T |
| 15136 | 0.0001 | 0.1249 | | 0.1577 | 0.029 | | 0.6883 | T |
| 15142 | 0.0001 | 0.7291 | | 0.0014 | 0.012 | | 0.2574 | A |
| 15145 | 0.0001 | 0.0089 | | 0.0042 | 0.955 | | 0.0318 | G |
| 15155 | 0.0002 | 0.0001 | | 0.0013 | 0.0001 | | 0.9982 | T |
| 15162 | 0.0001 | 0.0533 | | 0.0012 | 0.0002 | | 0.9451 | T |
| 15167 | 0.0003 | 0.1129 | | 0.0006 | 0.8857 | | 0.0004 | G |
| 15170 | 0.0101 | 0.0007 | | 0.9865 | 0.0013 | | 0.0013 | C |
| 15173 | 0.0101 | 0.0006 | | 0.8509 | 0.0002 | | 0.1381 | C |
| 15178 | 0.0117 | 0.9852 | | 0.0009 | 0.0013 | | 0.0009 | A |
| 15183 | 0.0181 | 0.0459 | | 0.0913 | 0.5127 | | 0.3321 | G |
| 15187 | 0.0752 | 0.0013 | | 0.9167 | 0.0015 | | 0.0052 | C |
| 15188 | 0.0748 | 0.7709 | | 0.0049 | 0.1462 | | 0.003 | A |
| 15189 | 0.0006 | 0.0668 | | 0.2123 | 0.2561 | | 0.4639 | T |
| 15190 | 0.0021 | 0.0499 | | 0.0628 | 0.3438 | | 0.5413 | T |
| 15191 | 0.0077 | 0.3002 | | 0.1278 | 0.0172 | | 0.5466 | T |
| 15192 | 0.0083 | 0.6135 | | 0.1339 | 0.0749 | | 0.169 | A |
| 15193 | 0.0101 | 0.3311 | | 0.0137 | 0.6341 | | 0.0108 | G |
| 15194 | 0.0803 | 0.0963 | | 0.0093 | 0.1357 | | 0.6782 | T |
| 15599 | 0.0748 | 0.0026 | | 0.1444 | 0.0043 | | 0.7737 | T |
| 15600 | 0.0751 | 0.0039 | | 0.0007 | 0.9174 | | 0.0029 | G |
| 15610 | 0.0098 | 0.1064 | | 0.0004 | 0.8822 | | 0.0012 | G |
| 15615 | 0.0097 | 0.0009 | | 0.0011 | 0.9872 | | 0.001 | G |
| 15621 | 0.0097 | 0.1425 | | 0.5372 | 0.3046 | | 0.006 | C |
| 15626 | 0.0098 | 0.9852 | | 0.0009 | 0.0015 | | 0.0025 | A |
| 15631 | 0.0084 | 0.0003 | | 0.9904 | 0.0001 | | 0.0007 | C |
| 15635 | 0.0002 | 0.001 | | 0.0062 | 0.0001 | | 0.9924 | T |
| 15640 | 0.0001 | 0.0058 | | 0.9557 | 0.0003 | | 0.0379 | C |
| 15644 | 0.0001 | 0.2136 | | 0.0132 | 0.0373 | | 0.7357 | T |
| 15650 | 0.0001 | 0.6657 | | 0.0283 | 0.1801 | | 0.1258 | A |
| 15655 | 0.0001 | 0.4699 | | 0.0103 | 0.4352 | | 0.0844 | A |
| 15663 | 0.0001 | 0.1944 | | 0.1678 | 0.505 | | 0.1326 | G |
| 15667 | 0.0001 | 0.0519 | | 0.029 | 0.7615 | | 0.1574 | G |
| 15670 | 0.0001 | 0.7208 | | 0.0011 | 0.1132 | | 0.1647 | A |
| 15675 | 0.0001 | 0.0493 | | 0.0103 | 0.8955 | | 0.0447 | G |
| 15683 | 0.0001 | 0.9978 | | 0.0001 | 0.0018 | | 0.0002 | A |
| 15690 | 0.0001 | 0.0001 | | 0.9993 | 0.0001 | | 0.0004 | C |
| 15694 | 0.0001 | 0.0031 | | 0.092 | 0.0001 | | 0.9046 | T |
| 15697 | 0.0001 | 0.0011 | | 0.0003 | 0.9982 | | 0.0001 | G |
| 15702 | 0.0001 | 0.0005 | | 0.9977 | 0.0001 | | 0.0015 | C |
| 15710 | 0.0004 | 0.0003 | | 0.9934 | 0.0002 | | 0.0058 | C |
| 15716 | 0.0002 | 0.1396 | | 0.0297 | 0.7861 | | 0.0444 | G |
| 15725 | 0.0002 | 0.0364 | | 0.0419 | 0.8586 | | 0.063 | G |
| 15729 | 0.0002 | 0.0096 | | 0.1045 | 0.2754 | | 0.6102 | T |
| 15748 | 0.102 | 0.0624 | | 0.0022 | 0.828 | | 0.0054 | G |
| 15774 | 0.0003 | 0.6724 | | 0.0046 | 0.1041 | | 0.2186 | A |
| 15798 | 0.0003 | 0.0099 | | 0.6082 | 0.0063 | | 0.3752 | C |
| 15828 | 0.0003 | 0.9892 | | 0.0002 | 0.0094 | | 0.0008 | A |
| 15861 | 0.0006 | 0.9963 | | 0.001 | 0.0014 | | 0.0007 | A |
| 15900 | 0.0002 | 0.5361 | | 0.2752 | 0.1787 | | 0.0098 | A |
| 15905 | 0.0001 | 0.0541 | | 0.6631 | 0.0514 | | 0.2312 | C |
| 15911 | 0.0001 | 0.0417 | | 0.7719 | 0.0322 | | 0.154 | C |
| 15922 | 0.0002 | 0.0032 | | 0.0002 | 0.9961 | | 0.0003 | G |
| 15927 | 0.0001 | 0.0007 | | 0.0001 | 0.9987 | | 0.0004 | G |
| 15937 | 0.0004 | 0.9972 | | 0.0001 | 0.002 | | 0.0002 | A |
| 15941 | 0.0004 | 0.0006 | | 0.0001 | 0.9987 | | 0.0001 | G |
| 15948 | 0.0001 | 0.0005 | | 0 | 0.9993 | | 0 | G |
| 15970 | 0.0002 | 0.9982 | | 0 | 0.0014 | | 0.0001 | A |
| 15980 | 0.0001 | 0.9944 | | 0.0001 | 0.0052 | | 0.0002 | A |
| 15990 | 0.0002 | 0.0004 | | 0.0001 | 0.9991 | | 0.0001 | G |
| 15997 | 0.0001 | 0.0011 | | 0.0001 | 0.9986 | | 0.0001 | G |
| 16005 | 0.0001 | 0.0113 | | 0.1007 | 0.0035 | | 0.8844 | T |
| 16022 | 0.0005 | 0.0022 | | 0.0001 | 0.9965 | | 0.0006 | G |
| 16025 | 0.0002 | 0.0158 | | 0.0007 | 0.9671 | | 0.0162 | G |
| 16029 | 0.0001 | 0.0012 | | 0 | 0.9986 | | 0.0001 | G |
| 16036 | 0.0001 | 0.0006 | | 0.0001 | 0.9991 | | 0.0001 | G |
| 16045 | 0.0001 | 0.9977 | | 0.0002 | 0.0017 | | 0.0003 | A |
| 16050 | 0.0001 | 0.0004 | | 0.1404 | 0.0039 | | 0.8551 | T |
| 16055 | 0.0001 | 0.0007 | | 0.0005 | 0.9982 | | 0.0005 | G |
| 16061 | 0.0001 | 0.9977 | | 0.0001 | 0.0019 | | 0.0002 | A |
| 16076 | 0.0001 | 0.0006 | | 0.9833 | 0.0021 | | 0.0139 | C |
| 16081 | 0.0001 | 0.0005 | | 0.0002 | 0.999 | | 0.0001 | G |
| 16085 | 0.0001 | 0.0001 | | 0.0019 | 0.0001 | | 0.9978 | T |
| 16093 | 0.0001 | 0 | | 0.9993 | 0 | | 0.0004 | C |
| 16101 | 0.0003 | 0.9966 | | 0.0002 | 0.001 | | 0.0017 | A |
| 16107 | 0.0001 | 0.9769 | | 0.0001 | 0.0227 | | 0.0001 | A |
| 16110 | 0.0001 | 0.5587 | | 0.0001 | 0.441 | | 0.0001 | A |
| 16114 | 0.0001 | 0.0001 | | 0.0012 | 0 | | 0.9985 | T |
| 16118 | 0.0002 | 0.0005 | | 0.9989 | 0 | | 0.0004 | C |
| 16125 | 0.0001 | 0.8534 | | 0.1443 | 0.0016 | | 0.0006 | A |
| 16131 | 0.0001 | 0.0002 | | 0.0014 | 0.0875 | | 0.9107 | T |
| 16139 | 0.0001 | 0 | | 0.9902 | 0.0003 | | 0.0093 | C |
| 16145 | 0.0001 | 0.9973 | | 0.0002 | 0.0022 | | 0.0002 | A |
| 16157 | 0.0001 | 0.0001 | | 0.0793 | 0.0001 | | 0.9203 | T |
| 16163 | 0.0001 | 0.0005 | | 0.0001 | 0.9975 | | 0.0017 | G |
| 16168 | 0.0004 | 0.0012 | | 0.6063 | 0.389 | | 0.003 | C |
| 16173 | 0.0002 | 0.0001 | | 0.9961 | 0.0002 | | 0.0034 | C |
| 16183 | 0.0001 | 0.0001 | | 0.9965 | 0.0002 | | 0.003 | C |
| 16187 | 0.0001 | 0.0001 | | 0.9388 | 0.0002 | | 0.0607 | C |
| 16197 | 0.0006 | 0.0034 | | 0.0037 | 0.0002 | | 0.9921 | T |
| 16210 | 0.0001 | 0.0005 | | 0.0013 | 0.0009 | | 0.9972 | T |
| 16215 | 0.0001 | 0.9988 | | 0.0001 | 0.0008 | | 0.0001 | A |
| 16222 | 0.0001 | 0.0007 | | 0.2739 | 0.0001 | | 0.7252 | T |
| 16238 | 0.0001 | 0.0515 | | 0.0001 | 0.9481 | | 0.0001 | G |
| 16245 | 0.0002 | 0.4337 | | 0.0588 | 0.1645 | | 0.3428 | A |
| 16249 | 0.0002 | 0.0021 | | 0.6531 | 0.1649 | | 0.1797 | C |
| 16258 | 0.0003 | 0.0044 | | 0.5878 | 0.0044 | | 0.403 | C |
| 16262 | 0.0002 | 0.3765 | | 0.0036 | 0.0839 | | 0.5358 | T |
| 16267 | 0.0001 | 0.0003 | | 0 | 0.9993 | | 0.0001 | G |
| 16270 | 0.0001 | 0.0004 | | 0.0001 | 0.9991 | | 0.0001 | G |
| 16282 | 0.0005 | 0.0005 | | 0.0001 | 0.9988 | | 0.0001 | G |
| 16289 | 0.0002 | 0.0002 | | 0.998 | 0.0007 | | 0.0008 | C |
| 16292 | 0.0002 | 0.0039 | | 0.0266 | 0.0333 | | 0.936 | T |
| 16345 | 0.0002 | 0.7592 | | 0.0005 | 0.0295 | | 0.2106 | A |
| 16349 | 0.0002 | 0 | | 0.9967 | 0.0026 | | 0.0004 | C |
| 16353 | 0.0003 | 0.9968 | | 0.0003 | 0.002 | | 0.0006 | A |
| 16361 | 0.0002 | 0.0099 | | 0.9887 | 0.0008 | | 0.0004 | C |
| 16372 | 0.0002 | 0.9034 | | 0.0005 | 0.0955 | | 0.0003 | A |
| 16375 | 0.0002 | 0.0001 | | 0.9978 | 0.001 | | 0.0008 | C |
| 16380 | 0.0002 | 0.1856 | | 0.0001 | 0.8139 | | 0.0001 | G |
| 16388 | 0.0002 | 0.0002 | | 0.0098 | 0.0001 | | 0.9897 | T |
| 16396 | 0.0002 | 0.0822 | | 0.0694 | 0.848 | | 0.0001 | G |
| 16401 | 0.0003 | 0.0941 | | 0.8494 | 0.0005 | | 0.0557 | C |
| 16407 | 0.0002 | 0.0007 | | 0.0012 | 0.0001 | | 0.9978 | T |
| 16411 | 0.0003 | 0.9978 | | 0.0004 | 0.0014 | | 0.0002 | A |
| 16419 | 0.0003 | 0.0001 | | 0.9991 | 0 | | 0.0005 | C |
| 16428 | 0.0003 | 0.9985 | | 0.0001 | 0.0009 | | 0.0001 | A |
| 16437 | 0.0004 | 0.9951 | | 0.0001 | 0.0021 | | 0.0024 | A |
| 16448 | 0.0003 | 0.0001 | | 0.0009 | 0.0001 | | 0.9985 | T |
| 16454 | 0.0004 | 0.0004 | | 0.0002 | 0.9989 | | 0.0001 | G |
| 16458 | 0.0004 | 0.0025 | | 0.0126 | 0.9838 | | 0.0007 | G |
| 16465 | 0.0005 | 0.2114 | | 0.4801 | 0.0791 | | 0.2289 | C |
| 16469 | 0.0005 | 0.1101 | | 0.4715 | 0.1969 | | 0.2209 | C |
| 16473 | 0.0006 | 0.2231 | | 0.0588 | 0.6149 | | 0.1025 | G |
| 16482 | 0.0022 | 0.2637 | | 0.0289 | 0.6213 | | 0.0838 | G |
| 16486 | 0.0005 | 0.0799 | | 0.0265 | 0.0888 | | 0.8042 | T |
| 16495 | 0.0005 | 0.9955 | | 0.0006 | 0.0027 | | 0.0006 | A |
| 16553 | 0.0005 | 0.0001 | | 0.9927 | 0.0001 | | 0.0066 | C |
| 16611 | 0.0005 | 0.9976 | | 0.0001 | 0.0015 | | 0.0001 | A |
| 16618 | 0.0006 | 0.5974 | | 0.0021 | 0.3997 | | 0.0001 | A |
| 16631 | 0.0007 | 0.7361 | | 0.1658 | 0.0012 | | 0.0961 | A |
| 16637 | 0.0005 | 0.0274 | | 0.0158 | 0.9549 | | 0.0013 | G |
| 16641 | 0.0007 | 0.2755 | | 0.0739 | 0.6365 | | 0.0133 | G |
| 16646 | 0.0006 | 0.0005 | | 0.0001 | 0.9988 | | 0.0001 | G |
| 16654 | 0.0007 | 0.2212 | | 0.3996 | 0.0101 | | 0.3683 | C |
| 16675 | 0.0006 | 0.4901 | | 0.1306 | 0.0866 | | 0.2919 | A |
| 16688 | 0.0137 | 0.004 | | 0.0057 | 0.9763 | | 0.0002 | G |
| 16691 | 0.0137 | 0.0006 | | 0.9719 | 0.0124 | | 0.0013 | C |
| 16697 | 0.0141 | 0.1935 | | 0.1449 | 0.5107 | | 0.1366 | G |
| 16719 | 0.0136 | 0.9621 | | 0.0054 | 0.0139 | | 0.0051 | A |
| 16736 | 0.0137 | 0.4324 | | 0.1775 | 0.2636 | | 0.1128 | A |
| 16738 | 0.0133 | 0.3966 | | 0.3818 | 0.1638 | | 0.0444 | A |
| 16743 | 0.0133 | 0.0242 | | 0.7624 | 0.0461 | | 0.1539 | C |
| 16747 | 0.0133 | 0.0861 | | 0.5761 | 0.134 | | 0.1903 | C |
| 16753 | 0.0133 | 0.0006 | | 0.0002 | 0.9856 | | 0.0002 | G |
| 16757 | 0.0133 | 0.0027 | | 0.655 | 0.0025 | | 0.3263 | C |
| 16766 | 0.0134 | 0.0783 | | 0.0002 | 0.9079 | | 0.0001 | G |
| 16775 | 0.0134 | 0.9844 | | 0.0001 | 0.0018 | | 0.0002 | A |
| 16814 | 0.0135 | 0.0439 | | 0.1336 | 0.7225 | | 0.0863 | G |
| 16818 | 0.0134 | 0.1226 | | 0.0449 | 0.7932 | | 0.0257 | G |
| 16824 | 0.0135 | 0.0269 | | 0.0796 | 0.3928 | | 0.4871 | T |
| 16829 | 0.014 | 0.0148 | | 0.3054 | 0.476 | | 0.1897 | G |
| 16833 | 0.0138 | 0.4081 | | 0.0108 | 0.5412 | | 0.0258 | G |
| 16836 | 0.0138 | 0.8465 | | 0.016 | 0.0111 | | 0.1125 | A |
| 16865 | 0.0135 | 0.001 | | 0.0007 | 0.9725 | | 0.0121 | G |
| 16871 | 0.0135 | 0.0003 | | 0.9755 | 0.0066 | | 0.0041 | C |
| 16876 | 0.0137 | 0.3111 | | 0.1162 | 0.2784 | | 0.2805 | A |
| 16881 | 0.0014 | 0.9441 | | 0.0005 | 0.0536 | | 0.0002 | A |
| 16884 | 0.0012 | 0.9975 | | 0.0001 | 0.001 | | 0.0002 | A |
| 16889 | 0.0012 | 0.0425 | | 0.0676 | 0.0033 | | 0.8854 | T |
| 16905 | 0.0012 | 0.0002 | | 0.9968 | 0.0001 | | 0.0017 | C |
| 16911 | 0.0071 | 0.0134 | | 0.4846 | 0.0741 | | 0.4207 | C |
| 16915 | 0.0381 | 0.0014 | | 0.9088 | 0.0156 | | 0.036 | C |
| 16919 | 0.0014 | 0.5827 | | 0.0679 | 0.1047 | | 0.2432 | A |
| 16920 | 0.1297 | 0.1942 | | 0.0681 | 0.2067 | | 0.4011 | T |
| 16928 | 0.0014 | 0.9965 | | 0.0004 | 0.0008 | | 0.0008 | A |
| 16934 | 0.0014 | 0.997 | | 0.0003 | 0.0011 | | 0.0001 | A |
| 16937 | 0.0014 | 0.9965 | | 0.0006 | 0.0014 | | 0.0001 | A |
| 16985 | 0.0039 | 0.2386 | | 0.0594 | 0.6408 | | 0.0572 | G |
| 16990 | 0.0019 | 0.0801 | | 0.6182 | 0.0777 | | 0.222 | C |
| 16994 | 0.0016 | 0.0993 | | 0.6015 | 0.0617 | | 0.2359 | C |
| 17002 | 0.0016 | 0.1649 | | 0.1774 | 0.5266 | | 0.1293 | G |
| 17005 | 0.0016 | 0.163 | | 0.0495 | 0.544 | | 0.2417 | G |
| 17011 | 0.0016 | 0.0007 | | 0.0764 | 0.0126 | | 0.9086 | T |
| 17021 | 0.0016 | 0.0002 | | 0.9965 | 0.0003 | | 0.0012 | C |
| 17030 | 0.0017 | 0.0118 | | 0.0963 | 0.1897 | | 0.7005 | T |
| 17037 | 0.0017 | 0.0159 | | 0.7427 | 0.0001 | | 0.2396 | C |
| 17042 | 0.0018 | 0.9935 | | 0.0002 | 0.0042 | | 0.0002 | A |
| 17048 | 0.0018 | 0.0011 | | 0.0003 | 0.9965 | | 0.0002 | G |
| 17054 | 0.0018 | 0.0002 | | 0.0017 | 0.0001 | | 0.9962 | T |
| 17064 | 0.0019 | 0.0021 | | 0.197 | 0.0016 | | 0.7973 | T |
| 17068 | 0.0019 | 0.0002 | | 0.9953 | 0.0015 | | 0.001 | C |
| 17072 | 0.002 | 0.0368 | | 0.0002 | 0.9609 | | 0.0001 | G |
| 17081 | 0.002 | 0.0021 | | 0.0001 | 0.9957 | | 0.0001 | G |
| 17152 | 0.002 | 0.9969 | | 0.0001 | 0.0009 | | 0.0001 | A |
| 17159 | 0.002 | 0.0005 | | 0.0648 | 0.0002 | | 0.9324 | T |
| 17163 | 0.0022 | 0.0043 | | 0.1586 | 0.0006 | | 0.8343 | T |
| 17166 | 0.0021 | 0.0026 | | 0.0002 | 0.9949 | | 0.0001 | G |
| 17170 | 0.0022 | 0.0373 | | 0.2091 | 0.3862 | | 0.3651 | G |
| 17178 | 0.0023 | 0.7605 | | 0.0068 | 0.2133 | | 0.0172 | A |
| 17182 | 0.0023 | 0.0005 | | 0.0769 | 0.9198 | | 0.0005 | G |
| 17189 | 0.0023 | 0.0008 | | 0.0074 | 0.2079 | | 0.7815 | T |
| 17197 | 0.0024 | 0.0003 | | 0.9942 | 0.0008 | | 0.0022 | C |
| 17202 | 0.0024 | 0.0005 | | 0.002 | 0.0001 | | 0.9949 | T |
| 17231 | 0.0025 | 0.0007 | | 0.0001 | 0.9965 | | 0.0001 | G |
| 17240 | 0.0026 | 0.0253 | | 0.9691 | 0.0008 | | 0.002 | C |
| 17249 | 0.0027 | 0.995 | | 0.0004 | 0.0016 | | 0.0002 | A |
| 17254 | 0.0029 | 0.9949 | | 0.0002 | 0.0018 | | 0.0002 | A |
| 17265 | 0.0029 | 0.0001 | | 0.988 | 0.0001 | | 0.0089 | C |
| 17275 | 0.0029 | 0.0003 | | 0.097 | 0.0001 | | 0.8997 | T |
| 17281 | 0.003 | 0.0002 | | 0.9948 | 0.0003 | | 0.0014 | C |
| 17296 | 0.0031 | 0.0014 | | 0.0003 | 0.9949 | | 0.0001 | G |
| 17300 | 0.0032 | 0.7748 | | 0.2081 | 0.0128 | | 0.0011 | A |
| 17304 | 0.0034 | 0.0007 | | 0.9176 | 0.0774 | | 0.0008 | C |
| 17308 | 0.0036 | 0.0171 | | 0.213 | 0.0069 | | 0.7594 | T |
| 17311 | 0.0037 | 0.3278 | | 0.3268 | 0.2458 | | 0.0958 | A |
| 17316 | 0.0037 | 0.0001 | | 0.9441 | 0.0002 | | 0.0518 | C |
| 17320 | 0.0038 | 0.8332 | | 0.0004 | 0.1585 | | 0.004 | A |
| 17326 | 0.0045 | 0.0004 | | 0.0656 | 0.0005 | | 0.929 | T |
| 17331 | 0.0041 | 0.0037 | | 0.0001 | 0.992 | | 0.0001 | G |
| 17339 | 0.0042 | 0.994 | | 0.0001 | 0.0015 | | 0.0002 | A |
| 17347 | 0.0042 | 0.9936 | | 0.0001 | 0.0019 | | 0.0001 | A |
| 17356 | 0.0045 | 0.0006 | | 0.0014 | 0.9933 | | 0.0002 | G |
| 17362 | 0.0047 | 0.0053 | | 0.3841 | 0.0356 | | 0.5704 | T |
| 17364 | 0.0046 | 0.0114 | | 0.5473 | 0.0005 | | 0.4359 | C |
| 17372 | 0.0051 | 0.0008 | | 0.0002 | 0.9938 | | 0.0002 | G |
| 17376 | 0.0051 | 0.0008 | | 0 | 0.9939 | | 0.0001 | G |
| 17379 | 0.0054 | 0.9927 | | 0.0001 | 0.0015 | | 0.0002 | A |
| 17388 | 0.0054 | 0.7165 | | 0.0002 | 0.2227 | | 0.0553 | A |
| 17393 | 0.0055 | 0.0003 | | 0.0012 | 0.0001 | | 0.9929 | T |
| 17400 | 0.0056 | 0.0005 | | 0.9149 | 0.0002 | | 0.0788 | C |
| 17404 | 0.0057 | 0.0139 | | 0.0004 | 0.9798 | | 0.0002 | G |
| 17409 | 0.0059 | 0.0009 | | 0.9901 | 0.0017 | | 0.0013 | C |
| 17419 | 0.0061 | 0.0003 | | 0.0011 | 0.0002 | | 0.9922 | T |
| 17432 | 0.0066 | 0.9916 | | 0.0001 | 0.0013 | | 0.0003 | A |
| 17442 | 0.0069 | 0.0005 | | 0.0001 | 0.9924 | | 0.0001 | G |
| 17456 | 0.0072 | 0.0001 | | 0.0008 | 0.0001 | | 0.9916 | T |
| 17471 | 0.0077 | 0.9913 | | 0.0001 | 0.0007 | | 0.0002 | A |
| 17475 | 0.0076 | 0.991 | | 0.0001 | 0.0011 | | 0.0002 | A |
| 17478 | 0.0079 | 0.002 | | 0.0081 | 0.0003 | | 0.9817 | T |
| 17486 | 0.0084 | 0.0004 | | 0.9757 | 0.0113 | | 0.0042 | C |
| 17490 | 0.0089 | 0.0015 | | 0.0077 | 0.9814 | | 0.0003 | G |
| 17494 | 0.0094 | 0.0011 | | 0.7283 | 0.0086 | | 0.2525 | C |
| 17498 | 0.0098 | 0.4669 | | 0.0044 | 0.5155 | | 0.0034 | G |
| 17509 | 0.0103 | 0.108 | | 0.0769 | 0.7732 | | 0.0314 | G |
| 17512 | 0.0103 | 0.9406 | | 0.0001 | 0.0485 | | 0.0005 | A |
| 17527 | 0.0105 | 0.0112 | | 0.0013 | 0.0002 | | 0.9769 | T |
| 17557 | 0.0106 | 0.0003 | | 0.9885 | 0.0001 | | 0.0005 | C |
| 17568 | 0.0108 | 0.9866 | | 0.0002 | 0.002 | | 0.0003 | A |
| 17581 | 0.0112 | 0.0011 | | 0.0006 | 0.9858 | | 0.001 | G |
| 17594 | 0.0118 | 0.2173 | | 0.7697 | 0.0005 | | 0.0007 | C |
| 17599 | 0.0122 | 0.952 | | 0.0014 | 0.0081 | | 0.0262 | A |
| 17611 | 0.0129 | 0.0046 | | 0.2794 | 0.0025 | | 0.7006 | T |
| 17672 | 0.0131 | 0.0478 | | 0.0003 | 0.9386 | | 0.0002 | G |
| 17676 | 0.0136 | 0.0301 | | 0.7347 | 0.0778 | | 0.1438 | C |
| 17687 | 0.0138 | 0.0035 | | 0.51 | 0.0044 | | 0.4682 | C |
| 17697 | 0.0141 | 0.2442 | | 0.0087 | 0.7324 | | 0.0005 | G |
| 17704 | 0.0144 | 0.0003 | | 0.9761 | 0.0075 | | 0.0016 | C |
| 17715 | 0.0149 | 0.0026 | | 0.0113 | 0.971 | | 0.0001 | G |
| 17724 | 0.0161 | 0.001 | | 0.0004 | 0.9806 | | 0.0019 | G |
| 17735 | 0.0169 | 0.001 | | 0.0018 | 0.0007 | | 0.9795 | T |
| 17755 | 0.017 | 0.0011 | | 0.0001 | 0.9815 | | 0.0001 | G |
| 17761 | 0.0196 | 0.9787 | | 0.0001 | 0.0014 | | 0.0002 | A |
| 17767 | 0.018 | 0.9799 | | 0.0001 | 0.0017 | | 0.0003 | A |
| 17773 | 0.0188 | 0.0029 | | 0.001 | 0.0005 | | 0.9769 | T |
| 17779 | 0.0194 | 0.9367 | | 0.0093 | 0.0295 | | 0.0049 | A |
| 17785 | 0.0202 | 0.0082 | | 0.9635 | 0.0008 | | 0.0073 | C |
| 17802 | 0.0209 | 0.0048 | | 0.0004 | 0.9733 | | 0.0005 | G |
| 17808 | 0.0216 | 0.0041 | | 0.0016 | 0.0006 | | 0.9721 | T |
| 17814 | 0.0223 | 0.0021 | | 0.0019 | 0.0007 | | 0.9729 | T |
| 17822 | 0.0225 | 0.001 | | 0.9748 | 0.0008 | | 0.0008 | C |
| 17827 | 0.0227 | 0.0008 | | 0.9504 | 0.0005 | | 0.0256 | C |
| 17878 | 0.0236 | 0.0004 | | 0.9692 | 0.0005 | | 0.0062 | C |
| 17886 | 0.0241 | 0.0099 | | 0.0006 | 0.965 | | 0.0002 | G |
| 17890 | 0.0246 | 0.0065 | | 0.0005 | 0.9682 | | 0.0001 | G |
| 17896 | 0.0271 | 0.0081 | | 0.0016 | 0.9624 | | 0.0006 | G |
| 17901 | 0.0275 | 0.0004 | | 0.5663 | 0.0635 | | 0.3421 | C |
| 17913 | 0.0299 | 0.0023 | | 0.9605 | 0.0024 | | 0.0044 | C |
| 17923 | 0.036 | 0.0008 | | 0.0024 | 0.0008 | | 0.9599 | T |

^1^ Position refers to the nucleotide position within the gapped alignment of the 962,279 bacterial *16S* rRNA gene sequences, and does not reflect the actual nucleotide position on the *16S* locus.

^2^ Nucleotide refers to the percentage, expressed as a decimal point, of each base, A (adenine), C (Cytosine), G (Guanine), and T (Tyrosine) at each position on the consensus sequence. Nucleotide frequencies higher than >90% are highlighted in Red.

^3^ Majority refers to the nucleotide that is most commonly found at this position across all 962,279 bacterial *16S* rRNA gene sequences. Nucleotide frequencies >90% are highlighted in Green, and the position of the *16S* rRNA real-time PCR primers are indicated with red arrows.
