# Supplementary material for: Detection of Bacterial 16S rRNA and Identification of Four Clinically Important Bacteria by Real-Time PCR
Source: PLoS One. 2012 Nov 6;7(11):e48558. doi: 10.1371/journal.pone.0048558 (PMC3490953; doi:10.1371/journal.pone.0048558)
Supplement: Table S2 — List of assembled E. coli genomes used in the design of E.coli -specific primers. (DOCX) [file pone.0048558.s002.docx]

**Supplemental Table S2. List of assembled *E. coli* genomes used in the design of *E.coli*-specific primers**

| **Designation** | **GenBank accession** |
| --- | --- |
| *Escherichia coli* str. K-12 substr. MG1655 | NC_000913.2 |
| *Escherichia coli* O157:H7 str. EDL933 | NC_002655.2 |
| *Escherichia coli* O157:H7 str. Sakai | NC_002695.1 |
| *Escherichia coli* UTI89 | NC_007946.1 |
| *Escherichia coli* 536 | NC_008253.1 |
| *Escherichia coli* APEC O1 | NC_008563.1 |
| *Escherichia coli* HS | NC_009800.1 |
| *Escherichia coli* E24377A | NC_009801.1 |
| *Escherichia coli* ATCC 8739 | NC_010468.1 |
| *Escherichia coli* str. K-12 substr. DH10B | NC_010473.1 |
| *Escherichia coli* SMS-3-5 | NC_010498.1 |
| *Escherichia coli* O157:H7 str. EC4115 | NC_011353.1 |
| *Escherichia coli* SE11 | NC_011415.1 |
| *Escherichia coli* O127:H6 str. E2348/69 | NC_011601.1 |
| *Escherichia coli* IAI1 | NC_011741.1 |
| *Escherichia coli* S88 | NC_011742.1 |
| *Escherichia coli* 55989 | NC_011748.1 |
| *Escherichia coli* IAI39 | NC_011750.1 |
| *Escherichia coli* UMN026 | NC_011751.1 |
| *Escherichia coli* LF82 | NC_011993.1 |
| *Escherichia coli* BW2952 | NC_012759.1 |
| *Escherichia coli* B str. REL606 | NC_012967.1 |
| *Escherichia coli* O157:H7 str. TW14359 | NC_013008.1 |
| *Escherichia coli* O103:H2 str. 12009 | NC_013353.1 |
| *Escherichia coli* O26:H11 str. 11368 | NC_013361.1 |
| *Escherichia coli* O111:H- str. 11128 | NC_013364.1 |
| *Escherichia coli* SE15 | NC_013654.1 |
| *Escherichia coli* DH1 | NC_017625.1 |
| *Escherichia coli* 042 | NC_017626.1 |
| *Escherichia coli* IHE3034 | NC_017628.1 |
| *Escherichia coli* ABU 83972 | NC_017631.1 |
| *Escherichia coli* ED1a | NC_017633.1 |
| *Escherichia coli* O83:H1 str. NRG 857C | NC_017634.1 |
| *Escherichia coli* NA114 | NC_017644.1 |
| *Escherichia coli* O7:K1 str. CE10 | NC_017646.1 |
| *Escherichia coli* O55:H7 str. CB9615 | NC_017656.1 |
| *Escherichia coli* KO11FL | NC_017660.1 |
| *Escherichia coli* P12b | NC_017663.1 |
