# Supplementary material for: Detection of Bacterial 16S rRNA and Identification of Four Clinically Important Bacteria by Real-Time PCR
Source: PLoS One. 2012 Nov 6;7(11):e48558. doi: 10.1371/journal.pone.0048558 (PMC3490953; doi:10.1371/journal.pone.0048558)
Supplement: Table S3 — Complete and draft A. baumannii genomes used the design of Acinetobacter -specific primers. (DOCX) [file pone.0048558.s003.docx]

**Supplemental Table S3. Complete and draft *A. baumannii* genomes used the design of *Acinetobacter*-specific primers**

| **Strain** | **Accession #^1^** | **Status^2^** |
| --- | --- | --- |
| 1656-2 | CP001921.1 | complete |
| AB0057 | NC_011586.1 | complete |
| AB307-0294 | NC_011595.1 | complete |
| ATCC 17978 | NC_009085.1 | complete |
| AYE | NC_010410.1 | complete |
| MDR-JZ06 | CP001937.1 | complete |
| SDF | NC_010400.1 | complete |
| TCDC-AB0715 | CP002522.2 | complete |
| 3909 | AEOZ01000001.1 | draft |
| 3990 | AEOY01000001.1 | draft |
| 4190 | AEPA01000001.1 | draft |
| 6013113 | ACYR01000001.1 | draft |
| 6013150 | ACYQ01000001.1 | draft |
| 6014059 | ACYS01000001.1 | draft |
| AB056 | ADGZ01000958.1 | draft |
| AB058 | ADHA01001039.1 | draft |
| AB059 | ADHB01001061.1 | draft |
| AB210 | AEOX01000020.1 | draft |
| AB900 | ABXK01000001.1 | draft |
| ABNIH1 | AFSZ00000000.1 | draft |
| ABNIH2 | AFTA00000000.1 | draft |
| ABNIH3 | AFTB00000000.1 | draft |
| ACICU | AFTC00000000.1 | draft |
| AL1 | unpublished | draft |
| AL23 | unpublished | draft |
| AL9 | unpublished | draft |
| ATCC 19606 | ACQB00000000.1 | draft |
| D1279779 | AERZ00000000.1 | draft |
| MDR-TJ | AEOE00000000.1 | draft |
| MRSN 4106 | unpublished | draft |
| MRSN 58 | unpublished | draft |
| MRSN 6272 | unpublished | draft |
| MRSN 6273 | unpublished | draft |
| MRSN 848 | unpublished | draft |
| MRSN 849 | unpublished | draft |
| MRSN 853 | unpublished | draft |
| MRSN 854 | unpublished | draft |
| MRSN 856 | unpublished | draft |
| MRSN 858 | unpublished | draft |
| MRSN 859 | unpublished | draft |
| MRSN 860 | unpublished | draft |
| MRSN 877 | unpublished | draft |
| MRSN 899 | unpublished | draft |
| MRSN 903 | unpublished | draft |
| MRSN 906 | unpublished | draft |
| MRSN 907 | unpublished | draft |
| MRSN 930 | unpublished | draft |
| MRSN 939 | unpublished | draft |
| MRSN 941 | unpublished | draft |
| MRSN 949 | unpublished | draft |
| MRSN 951 | unpublished | draft |
| MRSN 953 | unpublished | draft |
| MRSN 954 | unpublished | draft |
| MRSN 959 | unpublished | draft |
| MRSN 960 | unpublished | draft |
| MRSN 961 | unpublished | draft |
| UMB001 | AEPK01000002.1 | draft |
| UMB002 | AEPL01000002.1 | draft |
| UMB003 | AEPM01000002.1 | draft |
| WM99c | AERY00000000.1 | draft |

1 GenBank accession numbers for the completed or draft sequences are presented where available. The MRSN has access to a large number of draft *Acinetobacter* sequences that are currently not published.

2 Status indicates whether the genome has been completely annotated and published or whether just draft assemblies, consisting of multiple contigs are available.
