# Supplementary material for: Detection of Bacterial 16S rRNA and Identification of Four Clinically Important Bacteria by Real-Time PCR
Source: PLoS One. 2012 Nov 6;7(11):e48558. doi: 10.1371/journal.pone.0048558 (PMC3490953; doi:10.1371/journal.pone.0048558)
Supplement: Table S4 — The top 28 gene targets for designing Acinetobacter -specific primers. (DOCX) [file pone.0048558.s004.docx]

**Supplemental Table S4.** The top 28 gene targets for designing *Acinetobacter*-specific primers

| **gene product** | **HOGEMON family^1^** | **Length^2^** | **ACB variants^3^** | **best non-Acinetobacter match** | **Identity^4^** |
| --- | --- | --- | --- | --- | --- |
| segregation and condensation protein B | HBG752450 | 597 | 3 | none |  |
| preprotein translocase, SecE subunit | HBG701403 | 441 | 4 | none |  |
| 50S ribosomal protein L14 | HBG594899 | 369 | 1 | none |  |
| hypothetical protein | HBG634291 | 321 | 1 | none |  |
| enoyl-CoA hydratase/carnithine racemase | HBG340986 | 318 | 10 | none |  |
| hypothetical protein | HBG336893 | 288 | 9 | none |  |
| cell division topological specificity factor MinE | HBG449956 | 273 | 5 | none |  |
| hypothetical protein | HBG289877 | 246 | 3 | none |  |
| hypothetical protein | HBG289257 | 234 | 3 | none |  |
| translation initiation factor IF-1 | HBG286099 | 222 | 6 | none |  |
| hypothetical protein | HBG289808 | 219 | 6 | none |  |
| hypothetical protein | HBG638499 | 219 | 7 | none |  |
| hypothetical protein | HBG368435 | 213 | 9 | none |  |
| 50S ribosomal protein L29; 50S ribosomal protein L16 | HBG730819 | 198 | 2 | none |  |
| thiamine biosynthesis protein ThiS | HBG656610 | 198 | 6 | none |  |
| ribosomal protein L32 | HBG728381 | 186 | 3 | none |  |
| rubredoxin | HBG545811 | 165 | 2 | none |  |
| bacteriolytic lipoprotein entericidin B | HBG754046 | 144 | 4 | none |  |
| ribosomal protein L36 | HBG734088 | 117 | 3 | none |  |
| fructose-1,6-bisphosphate aldolase, class II | HBG327581 | 1038 | 10 | >gb\|ADOP01000046.1\| Pseudoalteromonas haloplanktis ANT/505 PH505bt | 669/899 |
| ribose-phosphate pyrophosphokinase | HBG519284 | 951 | 7 | >gb\|CP000083.1\|  Colwellia psychrerythraea 34H | 586/786 |
| Intracellular septation protein A | HBG663014 | 615 | 10 | >gb\|ABSQ01004398.1\|  Freshwater sediment metagenome lwMethenol_BCHA1873_x1 | 480/603 |
| 50S ribosomal protein L9 | HBG586587 | 447 | 4 | >gb\|CP001805.1\|  Vibrio sp. Ex25 chromosome 1 | 200/249 |
| ribosomal protein S12 | HBG747181 | 375 | 5 | >gb\|AFAK01000007.1\|  Succinivibrionaceae bacterium WG-1 contig00008 | 264/304 |
| ribosomal protein L7/L12 | HBG746222 | 372 | 5 | >gb\|ABYY01000008.1\|  Francisella philomiragia subsp. philomiragia ATCC 25015 cont1.8 | 205/267 |
| 50S ribosomal protein L19 | HBG611003 | 369 | 5 | >gb\|CP003488.1\|  Providencia stuartii MRSN 2154 | 175/218 |
| 50S ribosomal protein L23 | HBG439330 | 321 | 4 | >gb\|AACY021824069.1\|  Marine metagenome 1092343731685 | 292/322 |
| 30S ribosomal protein S15 | HBG500000 | 270 | 2 | Vibrio nigripulchritudo  ATCC 27043 VINI7043_2 | 151/183 |

1 HOGEMON family assignment is based on the definitions used by Penel and co-workers [17]

2 Gene length in bp

3 The number of nucleotide changes between all strains over the entire length of the gene. Optimal primer targets were selected based on the lowest number of nucleotide mismatches and overall length of the genes. The top three target genes are highlighted in Yellow

4 The number of nucleotides matching the sequence in the target gene based on a BLASTN analysis performed against the NCBI non-redundant nucleotide database.
