# Supplementary material for: Detection of Bacterial 16S rRNA and Identification of Four Clinically Important Bacteria by Real-Time PCR
Source: PLoS One. 2012 Nov 6;7(11):e48558. doi: 10.1371/journal.pone.0048558 (PMC3490953; doi:10.1371/journal.pone.0048558)
Supplement: Table S5 — Bacterial species used for specificity testing of species-specific primers. (DOCX) [file pone.0048558.s005.docx]

**Supplemental Table S5.** Bacterial species used for specificity testing of species-specific primers

| **Strain** | | **Designation^1^** |
| --- | --- | --- |
| *Acinetobacter* | *baumannii* | ATCC 19606 |
| *Acinetobacter* | *lwoffi* | Clinical isolate |
| *Achromobacter* | *xylosoxidans* | Clinical isolate |
| *Aeromonas* | *hydrophilia* | Clinical isolate |
| *Aeromonas* | *veronii* | Clinical isolate |
| *Bacillus* | *subtilis* | ATCC 6633 |
| *Burkholderia* | *cepacia* | ATCC 25416 |
| *Citrobacter* | *freundii* | ATCC 8090 |
| *Citrobacter* | *koseri* | Clinical isolate |
| *Clostridium* | *difficile* | ATCC 43255 |
| *Enterobacter* | *aerogenes* | ATCC 13048 |
| *Enterobacter* | *cloacae* | ATCC 13047 |
| *Enterobacter* | *gergoviae* | Clinical isolate |
| *Escherichia* | *coli* | ATCC 35218 |
| *Escherichia* | *coli O157* | ATCC 43888 |
| *Enterococcus* | *casse* | Clinical isolate |
| *Enterococcus* | *faecalis* | ATCC 51299 |
| *Enterococcus* | *faecium* | Clinical isolate |
| *Enterococcus* | *gallinarium* | ATCC 24311 |
| *Haemophilus* | *influenzae* | ATCC 10211 |
| *Hafnia* | *alvei* | ATCC 51873 |
| *Klebsiella* | *oxytoca* | Clinical isolate |
| *Klebsiella* | *pneumoniae* | ATCC 138 |
| *Kluyvera* | *ascorbata* | Clinical isolate |
| *Micrococcus* | *luteus* | ATCC 53 |
| *Moraxella* | *osloensis* | ATCC 10973 |
| *Morganella* | *morganii* | Clinical isolate |
| *Neisseria* | *meningitidis* | ATCC 53415 |
| *Pasteurella* | *multocida* | Clinical isolate |
| *Proteus* | *mirabilis* | ATCC 12453 |
| *Pseudomonas* | *aeruginosa* | ATCC 27853 |
| *Pseudomonas* | *flourescens* | ATCC 13525 |
| *Pseudomonas* | *putida* | Clinical isolate |
| *Pseudomonas* | *stutzeri* | Clinical isolate |
| *Providencia* | *rettgeri* | Clinical isolate |
| *Providencia* | *stuartii* | MRSN 2154 |
| *Serratia* | *marcesens* | ATCC 43861 |
| *Salmonella* | *typhi* | ATCC 14028 |
| *Shigella* | *flexneri* | ATCC 12022 |
| *Staphylococcus* | *aureus* | BAA 976 |
| *Staphylococcus* | *capitis* | Clinical isolate |
| *Staphylococcus* | *hemolyticus* | Clinical isolate |
| *Staphylococcus* | *epidermidis* | ATCC 12228 |
| *Staphylococcus* | *saprophyticus* | ATCC 15305 |
| *Streptococcus* | *agalactiae* | ATCC 12380 |
| *Streptococcus* | *pyogenes* | ATCC 19615 |
| *Streptococcus* | *pneumoniae* | ATCC 4969 |
| *Streptococcus* | *sanguis* | ATCC 10556 |
| *Streptococcus* | *salivaius* | ATCC 13419 |
| *Stenotrophomonas* | *maltocida* | Clinical isolate |

^1^ Identification of clinical isolates to the species level was performed on three automated identification systems; the Vitek 2 (bioMerieux, Durham, NC), the BD Pheonix (Diagnostics Systems, Sparks, MD), and the Microscan Walkway (Siemens Healthcare Diagnostics Inc, Deerfield, IL),
